# Advances in All-Solid-State Lithium–Sulfur Batteries for Commercialization

**DOI:** 10.1007/s40820-024-01385-6

**Published:** 2024-04-15

**Authors:** Birhanu Bayissa Gicha, Lemma Teshome Tufa, Njemuwa Nwaji, Xiaojun Hu, Jaebeom Lee

**Affiliations:** 1 Research Institute of Materials Chemistry, Chungnam National University, Daejeon, 34134 Republic of Korea; 2 Institute of Fundamental Technological Research, Polish Academy of Sciences, 02-106 Warsaw, Poland; 3 School of Life Sciences, Shanghai University, 200444 Shanghai, People’s Republic of China; 4 Department of Chemistry, Chungnam National University, Daejeon, 34134 Republic of Korea

**Keywords:** All-solid-state lithium–sulfur batteries, Commercialization, Enhancement strategies, Solid-state electrolytes, Sulfur-based cathodes

## Abstract

Solid-state batteries are commonly acknowledged as the forthcoming evolution in energy storage technologies. Recent development progress for these rechargeable batteries has notably accelerated their trajectory toward achieving commercial feasibility. In particular, all-solid-state lithium–sulfur batteries (ASSLSBs) that rely on lithium–sulfur reversible redox processes exhibit immense potential as an energy storage system, surpassing conventional lithium-ion batteries. This can be attributed predominantly to their exceptional energy density, extended operational lifespan, and heightened safety attributes. Despite these advantages, the adoption of ASSLSBs in the commercial sector has been sluggish. To expedite research and development in this particular area, this article provides a thorough review of the current state of ASSLSBs. We delve into an in-depth analysis of the rationale behind transitioning to ASSLSBs, explore the fundamental scientific principles involved, and provide a comprehensive evaluation of the main challenges faced by ASSLSBs. We suggest that future research in this field should prioritize plummeting the presence of inactive substances, adopting electrodes with optimum performance, minimizing interfacial resistance, and designing a scalable fabrication approach to facilitate the commercialization of ASSLSBs.

## Highlights

- Challenges in developing practical all-solid-state lithium–sulfur batteries (ASSLSBs) and recently devised concepts to address those critical challenges have been discussed.
- Recent developments in comprehending solid-state electrolytes, cathodes, and highperformance anodes, including key challenges associated with ion transport, electrochemical properties, and processing methods, have been discussed.
- Prospects of ASSLSBs for commercial use and guiding forthcoming research and development efforts in this area have been presented.

## Graphical abstract

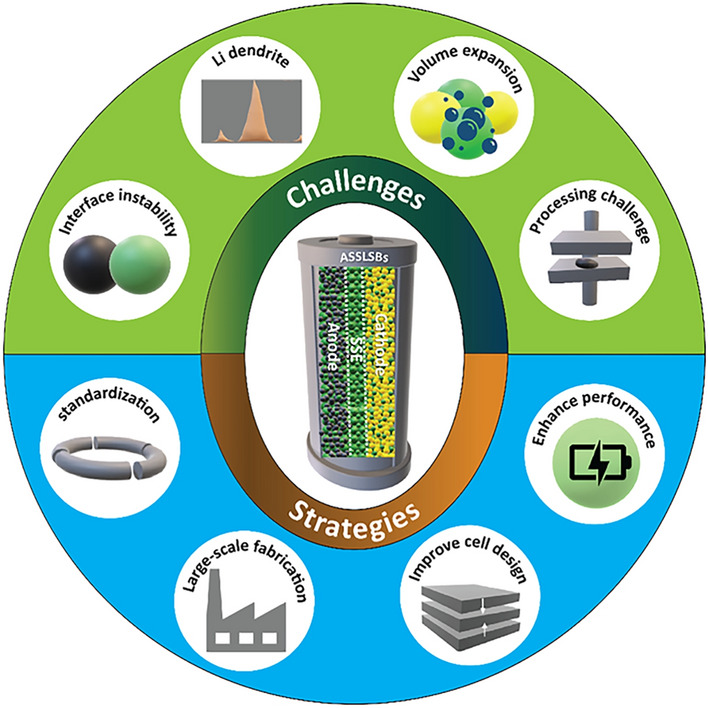

## Introduction

The swift progression of industry and the growing affluence contemporary civilization undercores the immense significance of establishing a sustainable and environmentally friendly economy. Despite the current consensus on low-carbon green growth, fossil fuels, including coal, oil, and natural gas, constituted over 80% of the global energy provision in 2021 [1]. The present condition and potential outcomes projected by the International Energy Agency (IEA) suggest that conventional sources of energy will persist in impacting the world’s energy system, while the proportion of renewable energy sources will gradually increase [2]. However, the use of fossil fuels has emitted substantial quantities of greenhouse gases (GHGs) that have led to a rise in the planet’s temperature and triggered ecological apprehensions. To deal with the energy and environmental issues linked to fossil fuel use, a new approach to energy and sustainable development has been proposed to reduce GHG emissions and develop renewable energy sources. Amidst the established strategies, a transition away from fossil fuels to low-carbon solutions is imperative for industries with high carbon emissions, specifically the transportation industry [3–5]. To enable this transition, it is crucial to develop technological advancements in the form of energy storage and conversion systems that can effectively store surplus renewable energy during periods of low consumption and supply it during periods of high consumption [6, 7].

There exists a vast range of energy storage technologies with significant differences in energy and power density, durability, effectiveness, cost, and other factors. When compared to alternative energy storage technologies, lithium-ion batteries (LIBs) have proven to offer a superior energy density and longer operating lifespan, making them the go-to option for energy storage in modern portable gadgets and electric vehicles (EVs) [8–10]. Since its inception, LIB has made remarkable progress in terms of material developments and electrode engineering, paving the way for their extensive utilization in diverse domains. Ever since Sony Corporation introduced the first product in 1991, LIBs energy density and specific energy have more than doubled from 120 Wh kg^−1^/264 Wh L^−1^ to 270 Wh kg^−1^/650 Wh L^−1^, while the real price in USD kW^−1^ h^−1^ has fallen nearly by 97% [11]. Although LIBs have been the preferred option thus far, there is a growing demand for increased capacity, improved safety, reduced costs, and sustained cyclability in various emerging applications.

One of the primary challenges of LIBs is their lower energy storage capability to meet growing demand [12]. The fundamental electrochemical combination of higher-energy designs in conventional LIBs has remained unchanged since their introduction. This stagnation has led to only minor enhancements in the nominal voltages of LIBs, posing challenges for applications that require higher-energy densities, such as EVs and renewable energy storage [13]. Currently, LIBs have a comparatively lower energy density in comparison to gasoline, with values ranging from 100 to 265 Wh kg^−1^ [14]. To meet the increasing demands of high-density energy storage systems like EVs, it is crucial to increase the energy density to at least 350 Wh kg^−1^ [15]. Furthermore, the electrochemical performance of LIBs declines considerably at low temperatures (< − 20 °C), resulting in notable losses of energy and power, charging difficulties, reduced lifespan, and safety concerns [16]. This is attributed to a significant decrease in the ability of ions to conduct electricity, slow transfer of charges at the interfaces, and sluggish movement of Li^+^ within the electrolyte interface and electrodes [17]. Additionally, the high activity of electrodes and the flammability of the organic electrolyte pose a significant safety risk [18]. In this regard, technological innovations and rational designs of electrolytes, such as the solidification of conventional flammable organic liquid electrolytes, are ideal solutions [19].

In particular, designing solid-state electrolytes (SSEs) characterized by elevated ionic conductivity and excellent formability at room temperature could prove to be a feasible solution for addressing safety concerns and enhancing the energy density of LIBs. Substituting flammable liquid organic electrolytes with SSEs has the potential to solve explosion and fire hazards, making ASSLSBs the most favorable option for use in EVs. ASSLSBs offer remarkable thermal resilience, capable of withstanding temperatures exceeding 200 °C, a feat unattainable by organic liquid electrolytes, which tend to evaporate beyond 70 °C, risking structural damage and exposing lithium metal. Owing to their high ionic conductivity and capacity to maintain low interfacial resistance during cycling, sulfide-based SSEs exhibit a competitive advantage, which is the primary challenge for almost all LIBs [20, 21]. Sulfide materials also offer significant benefits in processing due to their favorable mechanical characteristics and economical pricing [22]. These batteries promise to significantly enhance weight-to-energy density ratios, bolstering overall energy density and opening new avenues for commercial application. Moreover, the abundance of sulfur as a raw material compared to expensive heavy metals like cobalt and lithium makes ASSLSBs a cost-effective and sustainable option for next-generation batteries. Nevertheless, the notable disparity between fundamental scientific research and real-world implementation is a significant obstacle that has impeded the widespread adoption of ASSLSBs in commercial settings [23].

**Fig. 1:**
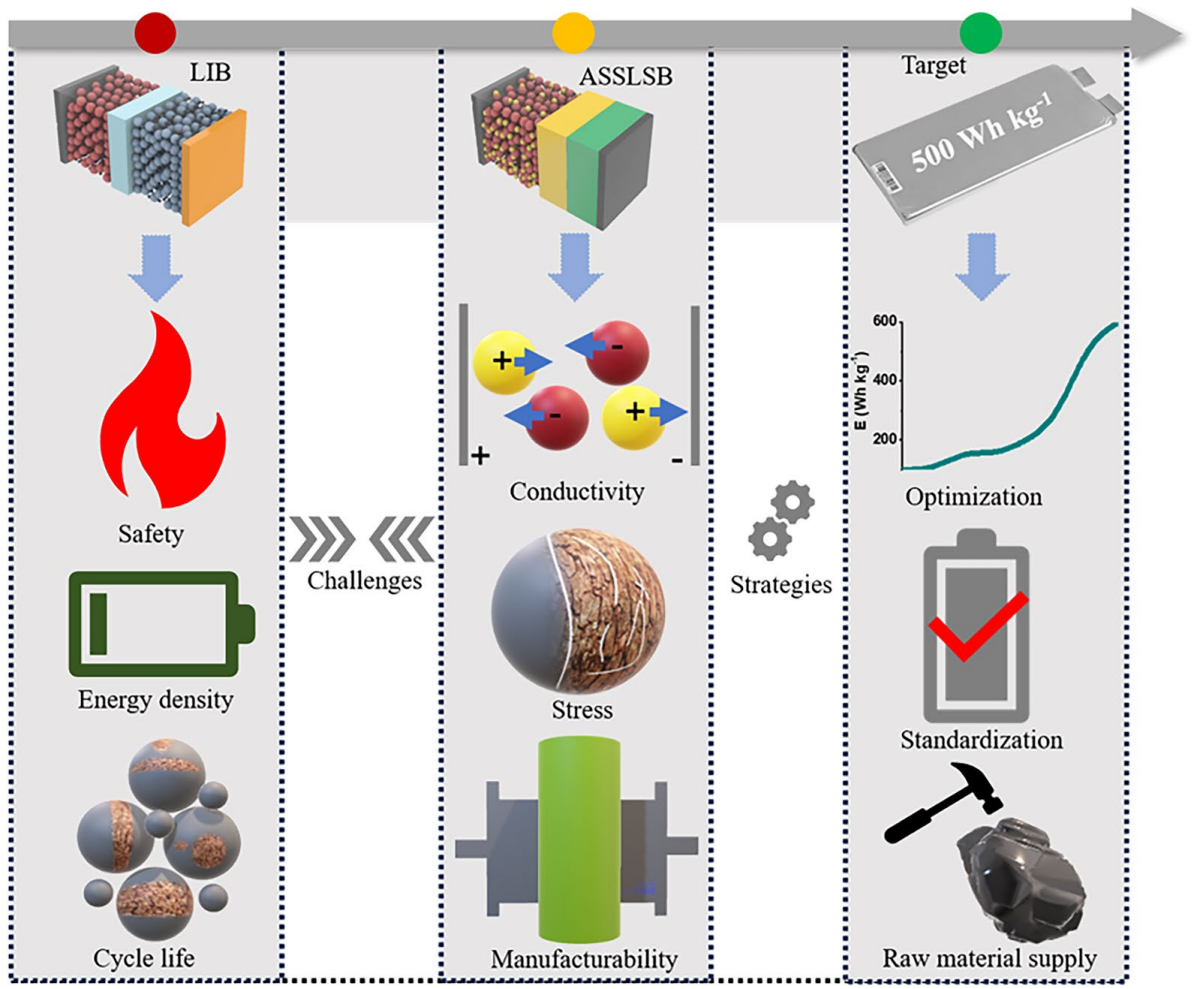
Summary of the various challenges of LIBs and ASSLSBs and corresponding strategies to accelerate the commercialization of ASSLSBs

This review specifically addresses the difficulties in developing functional ASSLSBs and presents an overview of newly formulated concepts to address these critical challenges (Fig. 1). We analyze and evaluate existing research in order to provide a comprehensive overview of the challenges, opportunities, and advancements in this field. We explore recent advances in comprehending the fundamental concepts of SSEs and cathodes, as well as high-performance anodes. We focus on addressing key challenges related to ion transport, electrochemical and mechanical properties, and recent processing methods. By addressing these issues, the article seeks to inform researchers, industry professionals, and academics about the prospects of ASSLSBs for commercial use and guide forthcoming research and development efforts in this area.

## Need for All-Solid-State Lithium–Sulfur Batteries

### Safety Concerns

The application of LIBs has grown in popularity in a diversity of sectors, such as EVs, electronics, and other expansive energy storage systems, in recent years. This trend is attributable to their extended lifespan, high energy density, and enhanced power storage capabilities [24]. However, it is important to acknowledge that these batteries also pose significant safety risks [25]. The electrodes within LIBs are highly reactive, and the electrolyte used in these batteries is flammable. Consequently, numerous incidents of explosions and fires associated with LIBs have occurred, leading to substantial financial losses for industries relying on these batteries and damaging the reputation of LIBs [26]. Recent incidents involving Boeing 787 Dreamliners, Tesla EV battery fires, and Samsung Note 7 explosions further highlight the critical importance of battery safety [27]. The safety of batteries is greatly influenced by their chemical composition, operating environment, and ability to withstand abuse [28]. Among these factors, thermal runaway is a major focus of battery safety research as it can result in disastrous failures of LIB systems [29, 30]. Exothermic reactions caused by breakdown processes between the electrolyte and active materials are generally believed to be the source of the energy released during thermal runaway. The organic liquid electrolyte inside LIBs is intrinsically flammable. The chain reaction of thermal runaway is triggered by side reactions occurring during battery operation and structural damage resulting from mechanical, electrical, or thermal abuse [25, 31].

**Fig. 2:**
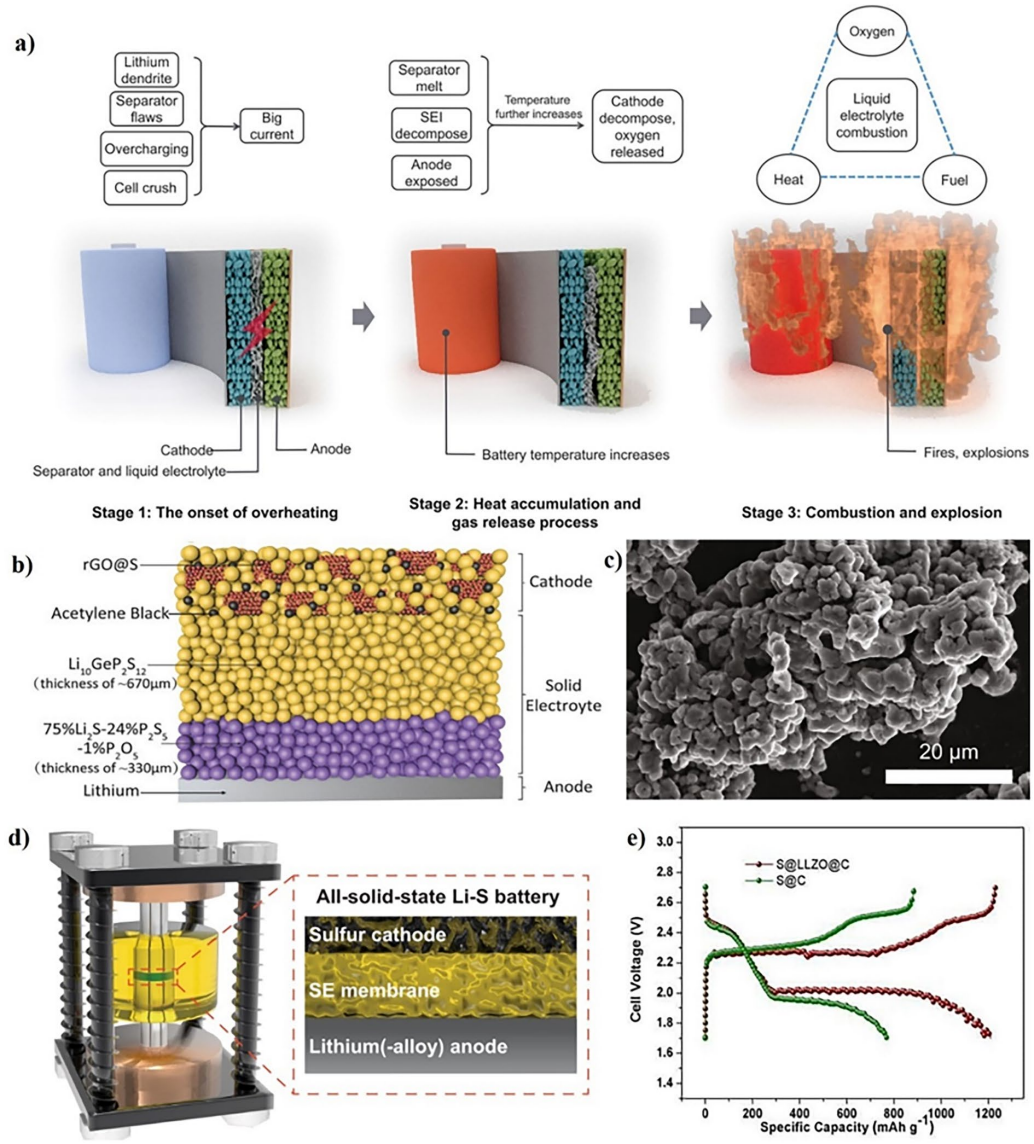
**a** Three stages delineate the thermal runaway process of LIBs. Copyright 2018, American Association for the Advancement of Science [34]. **b** Schematic diagram of an ASSLSB. Copyright 2017, WILEY–VCH [39]. **c** SEM image and **d** Schematic illustration of the Swagelok cell and the configuration of Li–S ASSBs. Copyright 2023, The Author(s), Springer Nature Limited [40]. **e** Typical charge/discharge curves of the S@LLZO@C and S@C cathodes with a current density of 0.1 mA cm.^−2^ at 50 °C. Copyright 2017, American Association for the Advancement of Science [41]

The thermal runaway process has been extensively discussed in various scientific reviews [32, 33]. The battery system can overheat if exposed to excessive heat, overcharged, experiences external or internal short circuits, or has a faulty cell. This can cause thermal runaway (Fig. 2a) [34]. Among these causes, internal shorting is the most common and can arise from issues like cell crushing, the formation of Li dendrites, and flawed separators during battery assembly. Notable examples include the Tesla car incident where the battery short-circuited and caught fire due to collision with metal debris, and the Samsung Note 7 battery fires caused by an ultrathin separator that was prone to damage [35]. Once the internal temperature begins to rise, a cascade of reactions takes place within the battery, ultimately leading to a device fire [36]. The initial step includes breaking down the solid electrolyte interphase (SEI) into stable components such as LiF and Li_2_CO_3_, as well as less stable elements like polymers, ROCO_2_Li, (CH_2_OCO_2_Li)_2_, and ROLi. These unstable components can breakdown at temperatures above 90 °C, causing a potentially deadly reaction that produces combustible gases and oxygen.

1 $$ \left( {{\text{CH}}_{{2}} {\text{OCO}}_{{2}} {\text{Li}}} \right)_{{2}} \to {\text{ Li}}_{{2}} {\text{CO}}_{{3}} + {\text{ C}}_{{2}} {\text{H}}_{{4}} + {\text{ CO}}_{{2}} + \, 0.{\text{5O}}_{{2}} $$

As the temperature rises, the organic solvents in the electrolyte come into contact with the Li metal or intercalated Li in the anode. This is what breaks down the SEIs.

2 $$ {\text{2Li }} + {\text{ C}}_{{3}} {\text{H}}_{{4}} {\text{O}}_{{3}} \left( {{\text{EC}}} \right) \, \to {\text{ Li}}_{{2}} {\text{CO}}_{{3}} + {\text{ C}}_{{2}} {\text{H}}_{{4}} $$

3 $$ {\text{2Li }} + {\text{ C}}_{{4}} {\text{H}}_{{6}} {\text{O}}_{{3}} \left( {{\text{PC}}} \right) \to {\text{Li}}_{{2}} {\text{CO}}_{{3}} + {\text{ C}}_{{3}} {\text{H}}_{{6}} $$

4 $$ {\text{2Li }} + {\text{ C}}_{{3}} {\text{H}}_{{6}} {\text{O}}_{{3}} \left( {{\text{DMC}}} \right) \, \to {\text{ Li}}_{{2}} {\text{CO}}_{{3}} + {\text{ C}}_{{2}} {\text{H}}_{{6}} $$

At temperatures exceeding 130 °C, polyethylene (PE) and polypropylene (PP) separators undergo a phase transition into a liquid state, intensifying the process by facilitating a connection between the anode and cathode, ultimately causing a faulty circuit. Moreover, the elevated temperature ultimately causes the disintegration of the cathode material, which is primarily composed of Li metal oxide, resulting in the release of oxygen. This disintegration process is highly exothermic, further aggravating the reactions.

5 $$ {\text{Li}}_{{\text{x}}} {\text{CoO}}_{{2}} \to {\text{ xLiCoO}}_{{2}} + { 1}/{3 }\left( {{1} - {\text{x}}} \right){\text{ Co}}_{{3}} {\text{O}}_{{4}} + { 1}/{3}\left( {{1} - {\text{x}}} \right){\text{O}}_{{2}} $$

6 $$ {\text{Co}}_{{3}} {\text{O}}_{{4}} \to {\text{ 3CoO }} + \, 0.{\text{5O}}_{{2}} $$

7 $$ {\text{CoO }} \to {\text{ Co }} + \, 0.{\text{5O}}_{{2}} $$

With the increasing demand for high-safety energy storage technologies, ASSLSBs that do not require organic liquid electrolytes have garnered considerable interest in recent years [37]. This approach circumvents the potential hazards associated with polymeric separators that are thermally unstable, and organic liquid electrolytes that are toxic and combustible. In comparison to LIBs and other potential beyond-LIB technologies utilizing organic liquid electrolytes, ASSLSBs are significantly more attractive for use in consumer electronics and EVs due to their inherent safety. Sulfide-type SSEs are perceived as the most promising electrolytes for ASSLSBs because of their compatibility with sulfur cathodes, strong ionic conductivity, and ease of processing and molding [38].

The integration of SSEs in ASSLSBs ensures exceptional thermal resilience, even in environments surpassing 200 °C [42]. Conversely, organic liquid electrolytes encounter severe challenges in high-temperature settings, as they tend to evaporate at temperatures exceeding 70 °C [43]. The vaporization causes the battery pack to expand and partially break, exposing the Li metal to air and risking a potential security breach. ASSLSBs are anticipated to impede Li metal dendritic growth as well, an issue that has greatly challenged LIBs [44]. Moreover, the inflexible physical barrier between the anode and cathode in SSEs could inhibit unwanted redox reactant exchange, potentially leading to capacity loss and an internal short circuit. To this extent, several prominent automobile companies such as Toyota, Volkswagen, General Motors, Hyundai, and Ford have heavily invested in solid-state battery tech firms to achieve complete commercial implementation by the initial half of the twenty-first century [45].

### Growing Demand for Higher-Energy–Density Batteries

LIBs have achieved remarkable success in powering various devices. This accomplishment may be due to the identification of ideal materials for battery components as well as advancements in the manufacturing process [46]. The energy density of cylindrical LIBs used in consumer electronics has grown around 6% per year since mass production started. However, the demand for higher energy density is on the rise, and current LIBs cannot keep up, especially in smart grid and automotive applications [47]. Currently, the application of LIBs technology is restricted to cells that possess volumetric energy densities of no more than 650 Wh L^−1^ for volumetric energy densities and 250 Wh Kg^−1^ for gravimetric energy densities [48]. Unlike consumer electronics, automotive and smart grid applications demand advanced, high-energy–density batteries. Researchers are actively exploring “post-Li-ion” solutions to the issue of LIB energy density, which is a growing concern. Due to their increased gravimetric energy density and economic significance, ASSLSBs provide alternative avenues for enhancing the energy density of cutting-edge batteries [49]. Particularly, there is a strong research interest in sulfur-based ASSLSBs due to numerous benefits, such as the intrinsic high energy found in the chemistry of Li–S and the heightened energy efficiency achieved by eliminating the polysulfide shuttle [50, 51]. Furthermore, SSEs can charge quickly without electrolyte polarization since their Li-ion transference number is close to one [52]. Consequently, the anticipated high power densities are achieved because S has a much greater specific capacity of 1672 mAh g^−1^ [53]. It has the ability to significantly increase the weight-to-energy density of ASSLSBs, thereby enhancing the overall energy density when combined with a Li metal anode, opening up new possibilities for commercial use.

The development of sulfur-based ASSLSBs is anticipated to be the next significant step forward in energy storage, following the appearance of SSEs with ionic conductivities comparable to liquid electrolytes [54, 55]. For instance, Yao’s group presented a novel cathode developed by depositing nanoamorphous sulfur over reduced graphene oxide (rGO) to maintain improved electrical conductivity (Fig. 2b) [39]. The rGO@S nanocomposite is evenly distributed throughout the acetylene black and conductive Li_10_GeP_2_S_12_ composite material, enhancing the battery’s ability to maintain a stable and reversible capacity of 830 mAh g^−1^ at a rate of 1.0 C for 750 cycles. Using sulfur as the active material for the cathode and metallic Li as the active material for the anode may theoretically produce specific energy exceeding 900 Wh kg^−1^ [56]. For example, Wang’s group [40] has proven an approach to using SSEs with low density and strong ionic conductivity to achieve high specific capacity in sulfur-based ASSLSBs. The argyrodite glass–ceramic SSEs have enabled the creation of high-performance sulfur cathodes, as illustrated in Fig. 2c [40]. When combined with thin Li and SSEs membranes, this discovery has the potential to enable sulfur-based ASSLIBs to produce specific energies in excess of 300 Wh kg^−1^ (Fig. 2d) [40].

Progress in developing SSEs with better ionic conductivities should facilitate the use of ASSLSBs, especially those that use sulfur as the cathode component. Cui’s group has recently introduced sulfur-based ASSLIBs using PEO/LLZO composite polymer electrolytes and a sulfur cathode composed of S@LLZO@C [41]. These batteries have shown a promising capacity of 900 mAh g^−1^ (Fig. 2e**)** [41].

The specific energy density of the majority of ASSLSBs exceeds that of conventional LIBs, contingent upon the cathode weight containing sulfur (Table 1). It is expected that SSEs will streamline the process of unit assembly in large battery systems. For instance, in battery modules for cars that need to produce high voltage, liquid electrolytes necessitate the connection of numerous cells in series. This, in turn, requires the same amount of battery casings. Conversely, SSE systems offer serial communication through the sequential stacking of electrolyte layers and bipolar electrodes in a single battery container. This reduces the energy density relative to volume and weight, leading to a reduction in the weight and volume of battery casings. Additionally, a cooling system is crucial for preventing batteries from overheating. While this system takes up a significant volume in the battery module, it can be minimized or even eliminated in SSEs, contributing to an increase in energy density.

**Table 1:** Energy densities of recently reported ASSLSBs

Battery design [Cathode ∣Electrolyte ∣Anode]	Capacity [mAh g^−1^]	Average voltage [V]	Energy density [Wh kg^−1^]	References
MoS_6_-CNT20@15%Li_7_P_3_S_11_ ∣ Li_6_PS_5_Cl ∣ Li	1034.32		1640	[57]
Li_2_S ∣ PEO-based electrolytes ∣ Li	1133		416	[58]
LiCoO_2_ ∣ 78Li_2_S·22P_2_S_5_ ∣ In	112	3.1	10.9	[59]
LiCoO_2_ ∣ Li_3_PS_4_ ∣ In	150	3.1	11.4	[60]
F@NMC811∣Li_6_PS_5_Cl–Mg16Bi84 ∣ Li	200	4.3	310	[61]
LiCoO_2_∣ Li_10_GeP_2_S_12_∣ Graphite	104	2.2	14.6	[62]
S-3DG@SMC ∣ SMC ∣ Li	1680		588.8	[63]
S/PAN ∣ LCE ∣ Li	588	2.7	116	[64]
LiCoO_2_ ∣ Li_10_GeP_2_S_12_ ∣ In	112	3.1	19.2	[65]
LiCoO_2_ ∣ Li_10_GeP_2_S_12_ ∣ In	140	3.1	20.9	[66]
Co_3_S_4_ ∣ polydopamine-coated Li_6_PS_5_Cl ∣ Li	485.1		284.4	[67]
LiNi_1/3_Co_1/3_Mn_1/3_O_2_ ∣ Li_6_PS_5_Cl ∣ In	44	3.3	9.4	[68]
LiNi_1/3_Co_1/3_Mn_1/3_O_2_ ∣ Li_6_PS_5_Br ∣ In	109	3.3	27.0	[69]
LiNi_0.8_Co_0.1_Mn_0.1_O_2_ ∣ Li_3_PS_4_ ∣ In	124	3.1	22.5	[70]
LiNi_0.8_Co_0.15_Al_0.05_O_2_ ∣ 80Li_2_S·20P_2_S_5_ ∣ Graphite	120	3.7	40.0	[71]
LiNi_0.5_Mn_0.5_O_2_ ∣ 95(0.6Li_2_S·0.4SiS_2_)·5Li_4_SiO_4_ ∣ In	70	3.1	10.4	[72]
MoS_5_@10%graphene15%Li_7_P_3_S_11_∣Li_6_PS_5_Cl ∣ Li	570.7		470.3	[73]
FeS_2_ ∣ Na_3_PS_4_/Na_3_SbS_4_ ∣ Na_3_Sn	346	1.68	14.4	[74]
NaS_2_ ∣ Na_3_PS_4_ ∣ Na-Sn–C	869.2	1.68	8.1	[75]
NaS_2_ ∣ Na_3_PS_4_ ∣ Na-Sn–C	1050	1.68	11.8	[76]
S∣Li_3.25_Ge_0.25_P_0.75_S_4_∣Li–In	1200	1.68	18.9	[77]
LiCoO_2_ ∣ LiPVFM/LiODFB ∣ Li	133	3.7	359	[78]

### Raw Material Supplies and Sustainability Challenges

The utilization of storage technologies plays a significant role in the complete facilitation of renewable energy and promoting a shift from reliance on fossil fuels. Batteries are at the forefront of those technologies, particularly in sectors like consumer electronics and the electrification of transportation. The current prevalence of LIBs can be attributed to the technological progress and cost reductions achieved over the past few decades (Table 2). Despite the significant decrease in the cost of LIBs in recent years, manufacturing expenses are still restricted due to the requirement of expensive transition metals, including Co, Ni, and Mn, in the cathode in addition to Li [22]. Typical LIBs contain Li, Co, and Ni across the positive electrode and graphite in the negative electrode in addition to Al and Cu in various cell and pack constituents [79, 80].

**Table 2:** Prices of selected battery materials. Copyright 2023, IEA [82]

Year	Metal price (GBP)				
	Lithium carbonate	Cobalt	Nickel	Copper	Manganese
2015	45	80	153	92	103
2016	63	59	87	76	56
2017	100	100	100	100	100
2018	157	216	137	118	123
2019	106	92	125	103	112
2020	66	94	129	94	89
2021	65	111	178	131	95
2022	310	191	231	160	103
2023	563	131	305	153	92

In the past few years, there has been a substantial rise in the cost of the indispensable components that make up LIBs. The cost of Li has risen by approximately 300% in comparison to the rates observed in 2021, whereas Ni prices experienced an increase of over fourfold within a single day. This abrupt surge compelled the London Metal Exchange to suspend trading, marking the first occurrence of such an event in 3 decades [81]. The atypical deviations in the pricing trajectory of these materials result from a confluence of factors, including the escalating global demand for EVs, the ongoing sluggishness in the supply chain caused by the pandemic, and Russia’s persistent conflict in Ukraine, given that Russia is a significant global producer of Ni.

Moreover, Co reserves are highly concentrated in specific regions, and their extraction is frequently linked with socio-environmental challenges. The rising demand for EVs is putting a spotlight on certain materials like Co, graphite, and rare earths, which are extensively used in LIBs. Meanwhile, LIBs are approaching their inherent performance limits after experiencing constant capability improvement over the past few decades. To overcome those challenges, several alternative contenders for LIBs have been suggested, many of which were formulated with the objectives of cost-effectiveness and sustainability in mind [83]. These strategies encompass the utilization of charge carriers composed of ions derived from more economical elements, such as Na or Zn, in lieu of Li. Additionally, there is the substitution of environmentally detrimental conventional electrolytes with alternatives that are more ecologically benign, including aqueous or safe solid-state formulations, among various other possibilities [84, 85]. Following years of research and development, several types of post-LIBs are finally starting to see early commercial success. Among these, ASSLSBs arise as a promising option, as they present the possibility of replacing these scarce and expensive metals with easily accessible substances such as sulfur [86].

There are numerous benefits to the technological transition from LIB chemistry to ASSLSBs in relation to specific energies and costs. One of the most prevalent elements on earth is sulfur, making it an attractive choice for electrode materials in batteries. In comparison to the heavy metal-based Co, Mn compounds, and phosphates currently used in LIBs, sulfur is more affordable (0.1 $ kg^−1^ at current pricing, as opposed to 30 $ kg^−1^ for LiCoO_2_ at current prices) [87, 88]. For example, when considering the costs of active materials in Li–S batteries, the cost of Li is approximately 2.2 € per gram, and the cost of sulfur is around 0.04 € per gram. These numbers are comparable to the costs of active materials in LIBs, such as LiCoO_2_ at approximately 1.3 € per gram and LiFePO_4_ at approximately 1.3 € per gram. The cost of graphite, commonly used as an anode material in LIBs, is around 0.03 € per gram, while silicon (Si), an alternative anode material, is approximately 0.34 € per gram. This indicates that the energy stored per euro of active materials, expressed in terms of watt-hours per euro (Wh €AM^−1^), is much more advantageous for sulfur-based battery chemistry compared to the benchmark LIBs [89]. In a recent study, Li et al. [90] introduced a rechargeable flow battery that operates at room temperature. This novel battery employs cost-effective polysulfide anolytes in combination with either Li or Na and utilizes an oxygen or air cathode. The energy density of the solution surpasses that of current flow batteries, varying between 30 and 145 Wh L^−1^. Furthermore, the active material cost per unit of stored energy is remarkably minimal, estimated to be approximately US$1 (kWh)^−1^ in the case of Na polysulfide [90].

## Fundamentals of All-Solid-State Lithium–Sulfur Batteries

**Fig. 3:**
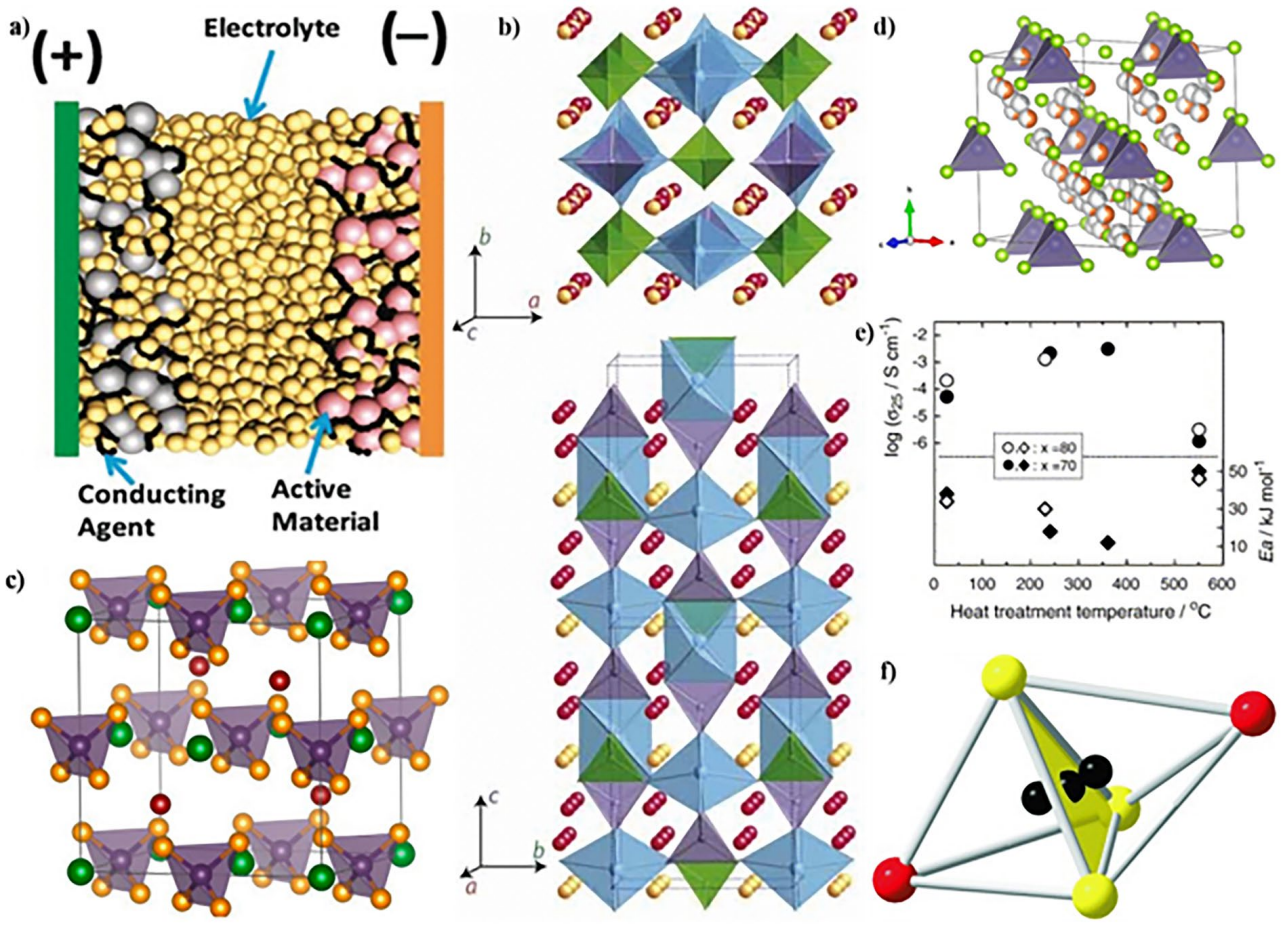
**a** Schematic illustrating the structure of an all-solid-state battery. Copyright 2021, American Chemical Society [91]. **b** Crystal structure of Li_10_GeP_2_S_12_. Copyright 2011, Springer Nature Limited [54]. **c** Unit cell of the Li_6_PS_5_X (X = Cl, Br, I), PS_4_^3–^ units in the octahedral interstices. Copyright 2021, American Chemical Society [99]. **d** Cubic crystal structure of Ag_8_GeSe_6_ at 473 K. Copyright 2021, American Chemical Society [100]. **e** Heat treatment temperature dependences of the ambient temperature conductivities (σ_25_) and activation energies (E_a_) for the xLi_2_S (100 − x) P_2_S_5_ (x = 70 and 80 mol%) glasses and glass–ceramics. Copyright 2006, Elsevier B.V. [101]. **f** A face-sharing S_3_I_2_ double tetrahedron. Copyright 2008, WILEY–VCH [102]

Solid-state batteries are composed entirely of solid components, as implied by their nomenclature. The fundamental contrast between conventional LIBs and ASSLSBs lies in replacing the liquid electrolyte with SSEs (Fig. 3a) [91]. Due to the flammability of organic solvents present in liquid electrolytes, conventional LIBs raise safety concerns, especially in high-power cell applications. As a result, the incorporation of non-combustible electrolytes in ASSLSBs improves their safety [92, 93]. Furthermore, SSEs exhibit intrinsic single-ion conductivity, indicating that Li transference and transport numbers are close to one. The presence of a single charge carrier reduces the influence of dynamic ion correlations, which might impair overall ionic conductivity within the bulk material [94]. Moreover, ASSLSBs have revived the possibility of using Li metal as an anode material instead of graphite [95].

The operational principle of ASSLSBs closely parallels that of conventional LIBs. During the discharge process, the cathode experiences reduction, while the anode undergoes oxidation. This coincides with the migration of Li ions through the SSEs from the anode to the cathode. During charge process, the migration of Li ions and electrons are reversed and the redox reaction can be described as S_8_ + 16 Li ↔ 8Li_2_S, with a voltage of roughly 2.15 V (vs. Li/Li^+^) [96]. A key advantage of ASSLSBs lies in the use of compact SSEs that act as physical barriers, inhibiting the formation of Li dendrites [97]. This property allows Li metal to be used as the anode material, which increases the volumetric energy density of LIBs by up to 70% compared to LIBs using graphite or other traditional anode materials [93]. Moreover, certain SSEs offer improved electrochemical stability, enabling the use of materials such as sulfur or high-voltage cathodes like LiNi_0.5_Mn_1.5_O_4_, known for their large capacity [98]. This results in increased energy densities at the cellular level. In typical LIBs, the liquid electrolyte connects every component of the battery cell, resulting in a parallel connection within the cell stack. This enables bipolar stacking, where a layer of Li^+^ isolation is used to connect individual cells in series. Implementing this arrangement increases the voltage of the battery cells while reducing the number of current collectors within the cell stack.

ASSLSBs have the benefit of not requiring a cooling system simply because they exclude flammable organic components. High temperatures, in fact, can improve their functioning by boosting conductance [103]. The ion migration kinetics in SSEs play a crucial role in the mass transport in advanced ASSLSBs. In contrast to conventional LIBs, SSEs exclusively contain a single type of mobile ion, Li^+^ [104]. Diffusion serves as a model for the movement of ions between various sites, including stable ground-state sites and metastable anion sites such as O^2−^, S^2−^, or polyanionic groups [96, 105]. Ion migration is influenced by the bonding environment of these sites, which is controlled by the arrangement and connectivity of anions.

There are three major pathways for ion movement in SSEs: (1) vacancy diffusion, where ions move to nearby unoccupied sites; (2) direct interstitial transmission, occurring between partially filled sites; and (3) coordinated or correlated interstitial systems, where interstitial ions transfer and compel nearby lattice ions to swap places with adjacent ions [106]. The ionic conductivity (*σ*) is a crucial parameter for describing ion transport in SSEs. *σ* is defined in the context of inorganic crystalline electrolytes by incorporating the charge (*q*), concentration (*n*), and charge carrier mobility (*υ*) into a modified Arrhenius equation:

8 $$ \sigma = { }qn\upsilon = \sigma_{0} T^{m} e^{{{\raise0.7ex\hbox{${ - E_{a} }$} \!\mathord{\left/ {\vphantom {{ - E_{a} } {k_{B} T}}}\right.\kern-0pt} \!\lower0.7ex\hbox{${k_{B} T}$}}}} $$

where *σ*_*0*_ denotes the intrinsic carrier density pre-exponential factor; *m* is commonly set to − 1; *k*_*B*_ represents the Boltzmann constant; *T* signifies the temperature; and *E*_*a*_ signifies the characteristic activation energy for ion conduction, which consists of the energy barrier for the migration of mobile defects (*E*_*m*_) and the formation energy of defects (*E*_*f*_).

### Sulfide Based Solid State Electrolytes

In the 1960s, when β-alumina was initially employed in Na–S batteries capable of withstanding high temperatures, SSEs had just begun their historical journey [107]. In the beginning, oxide SSEs were developed, but their low ionic conductivity hindered their application in ASSLSBs [108]. Sulfide-based electrolytes form weaker bonds with Li ions compared to oxide-based electrolytes, which is attributed to sulfur’s lower electronegativity and larger ionic radius than oxygen. This facilitates superior ion movement in sulfide electrolytes compared to oxide electrolytes, resulting in higher ion conductivity [67, 108]. Companies such as Toyota, Samsung SDI, CATL, and Solid Energy are diligently working to develop ASSLSBs, aiming to enhance the energy density and security of SSEs [109, 110].

#### Glasses

Due to its more open structure with larger spaces between particles, inorganic glass is commonly believed to have superior ionic conductivity compared to the same materials in crystal form (Fig. 3b) [54]. The binary system receiving the most attention is represented by xLi_2_S·(100 − x)P_2_S_5_, where x is the mole proportion. This system forms a single-phase glass between 0.4 and 0.8 [111]. Glass with a lower Li_2_S content (X ≤ 60) tends to have more di-tetrahedral P_2_S_7_^4−^ units, characterized by one S atom bridging and three S atoms on their own. Conversely, glass containing a higher concentration of Li_2_S (X ≤ 70) has a greater number of mono-tetrahedral P_2_S_7_^4−^ units, where all the S atoms are located at the ends. At room temperature, the 775Li_2_S·25P_2_S_5_ glass, entirely composed of PS_4_^3−^ units, exhibits a conductivity of 2.8 × 10^−4^ S cm^−1^ [112]. Another study discovered that 75Li_2_S·25P_2_S_5_ glass exhibits comparable ionic conductivities, falling within the range of 10^−4^ S cm^−1^ [113]. When the Li_2_S concentration exceeds 75, the decrease in crystallinity is likely caused by the presence of crystalline Li_2_S, which obstructs the conduction of Li^+^ ions [111]. Several binary glass systems with wide compositional ranges, including *x*Li_2_S·(100 − *x*)B_2_S_3_ and *x*Li_2_S·(100 − *x*)SiS_2_, have been synthesized and have ionic conductivities of around 104 S cm^−1^ at room temperature [114]. Conversely, xLi_2_S·(100 − x)GeS_2_ has exhibited lesser ionic conductivity in the range of about 10^−5^–10^−7^ S cm^−1^ [115].

Increasing the concentration and mobility of charge-carrying ions in SSE glass systems has the potential to enhance ionic conductivity [116]. One effective method for increasing the concentration of Li^+^ and improving conductivity is to dope the glass electrolytes with Li salts. For example, *x*Li_2_S·(100 − *x*)SiS_2_, with a large electrochemical window, doped with Li_3_PO_4_, Li_4_SiO_4,_ and Li_4_GeO_4_ displayed significantly increased conductivity over 10^−3^ S cm^−1^ [117]. Another successful approach is adding Li halides to the glass, as larger halide ions enhance ionic conductivity [118]. Ionic conductivities of about 10^−4^ S cm^−1^ are displayed by both *x*Li_2_S·(100 − *x*)B_2_S_3_ and *x*Li_2_S·(100 − *x*)SiS_2_ glass at ambient temperature [114, 119]. At ambient temperature, the ionic conductivity of a glass mixture containing 30Li_2_S·26B_2_S_3_·33LiI was 1.7 × 10^−3^ S cm^−1^, whereas a mixture containing 40Li_2_S·28SiS_2_·30LiI exhibited an ionic conductivity of 1.8 × 10^−3^ S cm^−11^ [120].

#### Crystalline Materials

##### Li–P–S Glassy Ceramics

In the LPS glass system, various sulfide crystals have been observed to form, including: Li_2_P_2_S_6_ (50Li_2_S·50P_2_S_5_) [121], Li_7_P_3_S_11_(70Li_2_S·30P_2_S_5_) [122], Li_3_PS_4_ (75Li_2_S·25P_2_S_5_) [123], Li_7_PS_6_ (88Li_2_S·12P_2_S_5_) [124] and Li_4_P_2_S_6_ (67Li_2_S·33P_2_S_5_) [125]. The specific formation of crystals is determined by both the glass composition and heat treatment parameters [112]. The creation of individual crystals with decreased ionic conductivity often results in a reduction in ionic conduction during glass crystallization. For instance, the formation of Li_4_P_2_S_6_, with a conductivity of approximately 10^−7^ S cm^−1^, significantly reduces the ionic conductivity of 67Li_2_S·33P_2_S_5_ glass substantially [101]. However, in the binary *x*Li_2_S·(100 − *x*)P_2_S_5_ system (*x* ≥ 70), high-temperature super-ionic metastable crystalline phases develop inside the glass components, resulting in an increase in ionic conductivity compared to the initial glass mix. In a separate investigation, researchers examined the 70Li_2_S·30P_2_S_5_ glass and glassy ceramics created at a temperature of 240 °C. At room temperature, the ionic conductivity of the original glass increased significantly from 5.4 × 10^−5^ to 3.2 × 10^−3^ S cm^−1^, as they observed during the creation of glass ceramics. Li_4_P_2_S_6,_ and Li_3_PS_4_, two crystalline phases with much lower ionic conductivity at 2.6 × 10^−8^ S cm^−1^, were detected when the same composition of 70Li_2_S·30P_2_S_5_ was applied in a solid-state manner. Therefore, solid-state reactivity is not a viable option for directly producing the super-ionic metastable phase; instead, glass crystallization is the only viable option [126].

At the composition of 75Li_2_S·25P_2_S_5_, the stoichiometric Li_3_PS_4_ phase precipitates as the ratio of Li_2_S increases [101]. Li_3_PS_4_ exhibits three different structures: the γ phase, which is at a low temperature; the β phase, which is at a medium temperature, and the α phase, which is at a high temperature. The conductivity of the γ-Li_3_PS_4_ phase, discovered in 1984, was 3 × 10^−7^ S cm^−1^ at 25 °C [127]. Both the γ and β phases possess an orthorhombic structure belonging to the *Pmn*21 space group. The PS_4_^3−^ tetrahedra in the γ phase are oriented in a particular manner, and Li atoms can be detected in two spots. The PS_4_^3−^ tetrahedra are packed in zigzag directions, with their apexes alternating in opposing orientations, and the β phase exhibits increased structural disarray (Fig. 3c) [99]. The same zigzag pattern is also observed in the α phase. The transition between phases increases the Li–S bond distance, and the thermally induced phase exhibits greater ionic conductivity due to its more favorable ionic conducting condition.

A significant increase in ionic conductivity, reaching approximately 3 × 10^−2^ S cm^−1^, is observed in the β phase of Li_3_PS_4_ at 227 °C [128]. At room temperature, a similar thio-LISICON III found in 78Li_2_S·22P_2_S_5_ glass was tuned to have a conductivity of 8.5 × 10^−4^ S cm^−1^ by controlling the rate of crystallization [129]. A newly generated phase, Li_7_P_2_S_8_I, with an even higher conductivity of 6.3 × 10^−4^ S cm^−1^ at 30 °C, emerges when LiI is added to the β-Li_3_PS_4_ phase. The superionic crystal Li_3.55_P_0.89_S_4_, also known as thio-LISICON II, initiates formation when the Li_2_S content reaches 80Li_2_S·20P_2_S_5_. This phase enhances the ionic conductivity to 1.3 × 10^−3^ S cm^−1^ at ambient temperature and has a monoclinic structure (Fig. 3d) [101]. At elevated temperatures, additional phases such as thio-LISICON III at 360 °C and Li_3.55_P_0.89_S_4_ at 550 °C form, whereas the stability of the thio-LISICON II phase extends only up to approximately 250 °C [101].

##### Li_6_PS_5_X (X = Cl, Br, and I) Argyrodite

Ag_8_GeS_6_ was the pioneering material discovered to possess a cubic argyrodite structure, featuring 136 tetrahedral sites per unit cell. Some of these positions are occupied by Ag^+^ and Ge^4+^ ions, resulting in an extremely disordered arrangement of cations [130]. Utilizing the same argyrodite structure, this material can incorporate Cu instead of Ag due to its high ionic conductivity and the mobility of Ag^+^ ions [131]. Based on this, Deiseroth et al. [102] proposed substituting Li^+^ ions for Ag^+^ ions and switching one halogen atom for another (Fig. 3e) [101]. The ionic conductivities of these compounds at room temperature are 1.9 × 10^−3^, 6.8 × 10^−4^, and 4.6 × 10^−7^ S cm^−1^, respectively [132]. The variation in ionic conductivity among these materials is attributed to the differing degrees of anion disorder [133, 134].

Minafra et al. [130] conducted a study where they replaced the P^5+^ ions in the Li_6_PS_5_Br argyrodite structure with Si^4+^ ions. The conductivity of the substituted material, Li_6.35_P_0.65_Si_0.35_S_5_Br, reached 2.4 × 10^−3^ S cm^−1^ at room temperature, marking a threefold improvement over the original material due to the enhanced coordinated mobility of Li^+^ ions. In related study, Kraft et al. [135] investigated the possibility of doping Li_6_PS_5_I with Ge^4+^ ions in a related study. However, they were unable to incorporate Ge^4+^ into Li_6_PS_5_Br or Li_6_PS_5_Cl because Ge^4+^ ions are rather big. Initially, Li_6_PS_5_I did not exhibit any site disorders. However, as the occupancy of Ge^4+^ on the P^5+^ site increased, disorder in the I^−^/S^2−^ site was observed at 20% Ge^4+^ replacement. As a result, Li was able to migrate through wider channels, leading to an increase in the volume of Li(48* h*)S_3_I tetrahedra and a decrease in the area of the Li(24* g*)S_3_ triangular plane. The current record for maximum ionic conductivity in the argyrodite family, achieved at room temperature by Li_6.6_P_0.4_Ge_0.6_S_5_I is 5.4 × 10^−3^ S cm^−1^ [136].

##### Thio-LISICONs

Thio-LISICON (Lithium SuperIonic CONductor) structures are observed in various systems (Fig. 3f) [102], such as Li_2_S–GeS_2_, Li_2_S–GeS_2–_ZnS, and Li_2_S–GeS_2_–Ga_2_S_3_ [102]. This structural motif is documented in six materials: Li_2_GeS_3_, Li_4_GeS_4_, Li_2_ZnGeS_4_, Li_4−2*x*_Zn_*x*_GeS_4_, Li_5_GaS_4_, and Li_4+*x*+*y*_(Ge_1−*y*−*x*_Ga_*x*_)S_4_. In these structures, S atoms form a densely arranged hexagonal pattern, heavy metal cations occupy tetrahedral sites, and Li atoms exhibit disorder within octahedral sites [137]. All of these materials demonstrate a voltage tolerance of up to 5 V when compared to the Li/Li^+^ reference electrode. The discovery of thio-LISICON structures has paved the way for synthesizing novel materials by utilizing PS_4_, SnS_4_, GeS_4_, and SiS_4_ tetrahedra as building blocks [137]. A notable example is Li_4_SnS_4_ (space group Pnma), sharing a crystal structure akin to Li_4_GeS_4_ and demonstrating an ionic conductivity of 7.0 × 10^−5^ S cm^−1^ at room temperature [138]. Furthermore, by aliovalent doping with arsenic (Li_3.833_Sn_0.833_As_0.166_S_4_), the conductivity of Li_4_SnS_4_ can be dramatically increased to 1.4 × 10^−3^ S cm^−1^ [139].

A novel set of crystalline thio-LISICON materials, denoted as Li_4−*x*_Ge_1−*x*_P_*x*_S_4−*x*_ (space group P2_1/m_), was synthesized by substituting Ge^4+^ with P^5+^ in Li_4_GeS_4_. This discovery emerged from the Li_2_S–GeS–P_2_S_5_ system. Analysis of structural properties through X-ray powder diffraction (XRD) revealed three distinct regions within the (1 − *x*)Li_4_GeS_4–*x*_Li_3_PS_4_ solid solution: the orthorhombic thio-LISICON I region (x ≤ 0.6), the monoclinic thio-LISICON II region (0.6 < x < 0.8), and the monoclinic thio-LISICON III region (x ≥ 0.8) [54]. The composition with x = 0.75, corresponding to Li_3.25_Ge_0.25_P_0.75_S_3.25_, exhibited the highest ionic conductivity among these regions at room temperature, reaching 2.2 × 10^−3^ S cm^−1^. These results suggest that Li_4−x_Ge_1−x_P_x_S_4−x_ materials hold promise as solid-state electrolytes (SSEs) for next-generation energy storage systems [54]. In a subsequent study, Hori et al. further explored these findings by constructing a more thorough phase diagram for the binary system of (1 − *x*)Li_4_GeS_4–*x*_Li_3_PS_4_ using XRD and differential thermal analysis [140]. The diffraction peaks observed in the study were identified as originating from three phases: Li_4_GeS_4_, Li_10_GeP_2_S_12_ (LGPS), and Li_3_PS_4_. The determined specific composition ranges for Li_4_GeS_4_, LGPS, and β-Li_3_PS_4_ were determined to be 0.1 ≤ *x* ≤ 0.2, 0.5 ≤ *x* ≤ 0.67, and 0.9 ≤ *x* ≤ 0.98, respectively. It is important to note that as the temperature increases, the solid solution range has the potential to widen. For instance, at 650 °C, a mixture of Li_4_GeS_4_ and LGPS was found to transform into a single-phase Li_4_GeS_4_ material. The observed temperature-dependent behavior offers insights into the synthesis and stability of thio-LISICON materials, indicating their potential applicability across various temperature regimes [140].

Modifications of Li_4_SiS_4_ can yield materials similar to those found in the Ge system. The polarizability and size of ions are crucial factors influencing the ionic conductivity of thio-LISICON materials. This is why the Ge-based compounds exhibit better conductivities compared to their Si counterparts. To avoid the use of rare and costly Ge, Al^3+^ can be substituted for Ge^4+^. This substitution eliminates non-bridging S and enhances ionic conductivity. As a result, Li_11_AlP_2_S_12_, an analog of thio-LISICON, has been the subject of study [141]. The sensitivity of phosphorus-containing sulfides to air and moisture was significant, leading to the use of Sn and As as principal components combined with S. The produced Li_4_SnS_4_ substance demonstrated remarkable stability in the air and exhibited an ionic conductivity of 7.1 × 10^−5^ S cm^−1^ at 25 °C. The total ionic conductivity is enhanced compared to the pure material when arsenic is used as a substitute for phosphorus, creating interstitials and/or vacancies in the crystal structure. The conductivity that was most impressively produced as a result of this replacement for Li_3.833_Sn_0.833_As_0.166_S_4_ reached an astounding value of 1.39 × 10^−3^ S cm^−1^ [139].

##### Li_11−x_M_2−x_P_1+x_S_12_ (M = Ge, Sn, and Si) Structures

To achieve high-energy–density ASSLSBs, it is imperative to develop a sulfide electrolyte with ultrahigh ionic conductivity. In 2011, Kamaya et al. [54] identified a tetragonal compound called Li_10_GeP_2_S_12_ (also referred to as LGPS) with a space group of *P*4_2_*/nmc*. The structure of LGPS is organized to form one-dimensional (1D) chains using (Ge_0.5_P_0.5_)S_4_ tetrahedra and LiS_6_ octahedra. LiS_4_ tetrahedra connect these chains, establishing a one-dimensional pathway for Li-ion conduction along the c-axis. The presence of tetrahedrally coordinated Li sites (16* h* and 8*f*) within the framework chains contributes to the creation of channels for Li conduction, whereas the octahedrally coordinated Li site (4*d*) remains inactive for conduction. A fourth Li site connecting the two active Li sites’ 1D diffusion channels was found by Kuhn et al. [142] using single crystal diffraction. They proposed that active diffusion takes place at this location, contributing to Li-ion conduction. Interestingly, Mo et al. [143] through first principle calculations, predicted that LGPS exhibits three-dimensional (3D) conductivity rather than being strictly confined to 1D. The significant Li hopping in the ab plane and the empty space between the (Ge_0.5_P_0.5_)S_4_ and LiS_4_ tetrahedra contribute to this behavior. The ab-plane diffusion of Li ions is relatively slower compared to the c-axis, and at ambient temperature, the anticipated ionic conductivity in this plane is 9.0 × 10^−4^ S cm^−1^. With an impressive ionic conductivity of 1.2 × 10^−2^ S cm^−1^ at ambient temperature, LGPS distinguishes itself as the first SSE to exhibit ionic conductivity equal to or even greater than that of liquid electrolyte. This property highlights the possibility of using LGPS in ASSLSBs and other sophisticated energy storage systems [144].

However, a notable drawback of LGPS materials is their high cost, primarily due to the use of Ge metal. As a result, further research has explored the possibility of replacing some or all of the Ge in the LGPS structure. Ong et al. [145] conducted an investigation and revealed that iso-valent cation substitutions of Ge^4+^ have a minimal impact on the diffusivity of Li ions within the tetragonal LGPS structure. This led to the emergence of Li_10_SnP_2_S_12_ (LSnPS) as an affordable alternative, featuring an isostructural arrangement similar to LGPS but with a slightly different disorder of Li ions. LSnPS shows a slightly higher resistance at grain boundaries when compared to LGPS, leading to a slightly lower overall ionic conductivity of approximately 4 × 10^−3^ S cm^−1^ at room temperature. However, the conductivity remains comparable to that of liquid electrolytes [146]. Bron et al. [147] achieved a significant improvement in ionic conductivity by substituting 30% of Sn with Si, leading to the formation of Li_10_Sn_0.7_Si_0.3_P_2_S_12_. This modification significantly reduced the resistance at grain boundaries, resulting in an elevated ionic conductivity of approximately 8 × 10^−3^ S cm^−1^ at room temperature. Another cost-effective alternative is Li_10_SiP_2_S_12_ (LSiPS), which adopts the crystalline LGPS structure with slightly smaller lattice parameters than LGPS (*a* = 8.65 Å and *c* = 12.51 Å for LSiPS, whereas *a* = 8.71 Å and *c* = 12.63 Å for LGPS). Ong et al. [145] reported that LSiPS exhibits a higher ionic conductivity of 2.3 × 10^−2^ S cm^−1^ compared to LGPS. However, experimental measurements indicate that LSiPS demonstrates a lower ionic conductivity of 2.3 × 10^−2^ S cm^−1^ at room temperature. This difference is likely attributed to the emergence of an orthorhombic bi-phase in LSiPS, introducing additional impedance recognized as Maxwell–Wagner type impedance [148]. Using 7Li nuclear magnetic resonance (NMR) techniques, Kuhn et al. [148] demonstrated that the Li-ion diffusivity in LSnPS is slightly lower than that in LGPS. To stabilize the tetragonal modification, a higher Si occupancy is required at the 4*d* site compared to Ge or Sn. This is why the Si analogue is obtained for the stoichiometry of Li_11_Si_2_PS_12_ rather than Li_10_SiP_2_S_12_ [149].

### Sulfide-Based Cathodes

#### Sulfur

**Fig. 4:**
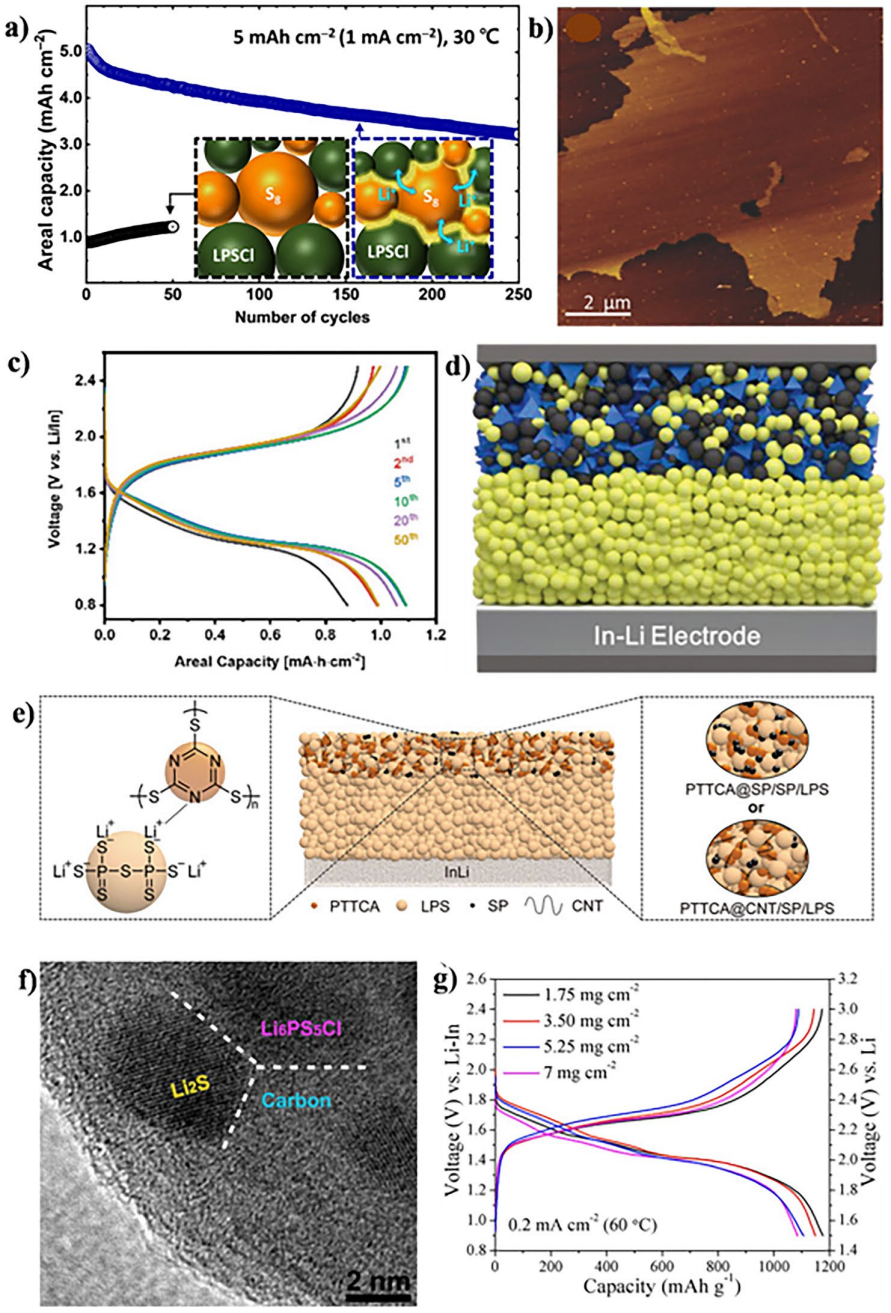
**a** A scheme of inorganic Li-ion-conducting species (3Li^+^-PS_4+*n*_^3–^ (*n* ≥ 0)) incorporated between S_8_ and the SSE of Li_6_PS_5_Cl (LPSCl) to enhance the ionic contact of S_8_. Copyright 2023, American Chemical Society [151]. **b** Atomic force microscopy (AFM) image of an amorphous rGO@S-40 composite on a Si substrate. Copyright 2017, WILEY–VCH [39]. **c** Electrochemical profile. Copyright 2020, WILEY–VCH [155]. **d** Schematic of the all-solid-state battery design for SnS nanocrystals. Copyright 2019, WILEY–VCH [156]. **e** Schematics of the ASSLSB with LPS electrolyte and poly (trithiocyanuric acid) PTTCA cathode (center), LPS-PTTCA interaction (left), and PTTCA@SP and PTTCA@CNT cathode topologies (right). Copyright 2021, WILEY–VCH [157]. **f** The high-resolution transmission electron microscopy (HRTEM) image of the as-obtained Li_2_S−Li_6_PS_5_Cl−C nanocomposite. Copyright 2016, American Chemical Society [158]. **g** Typical voltage profiles with areal Li_2_S loading from 1.75 to 7 mg cm^−2^. Copyright 2019, American Chemical Society [178]

Due to its cost-effectiveness and a high theoretical specific capacity of 1675 mAh g^−1^, elemental S emerges as a highly attractive active ingredient for ASSLSBs [150]. Additionally, the chemical compatibility between S and sulfide SSEs at the interface is advantageous for ASSLSBs (Fig. 4a) [151]. It is noteworthy that S materials were not exclusively used as the cathode active material in the earliest phases of ASSLSB development. To enhance electronic conductivity, composite cathodes were integrated with metallic Cu. Hayashi et al. [152] observed significant performance fluctuations in ASSLSBs, correlating with varying molar ratios of S/Cu and the duration of mechanical ball milling. The battery with a cathode material featuring an S/Cu ratio of 3, subjected to 15 min of milling, exhibited optimal electrochemical performance. This battery showed a discharge capacity greater than 650 mAh g^−1^ over the course of 20 cycles. XRD findings revealed the production of CuS through the ball-milling procedure, subsequently serving as an active constituent in the battery. The combination of S with metallic Cu, along with the optimization of the S to Cu ratio by researchers, elevated the electrochemical performance and electronic conductivity of ASSLSBs. These devices utilized both CuS and S as active materials.

Subsequent research has documented studies on ASSLSBs utilizing elemental S as the cathode active material [153, 154]. A notable advancement in this field was documented by Yao et al. [39] in 2017. The researchers deposited amorphous S onto rGO, a conductive material, as part of their investigation (Fig. 4b) [47]. The rGO@S composites were subsequently uniformly dispersed throughout the LGPS electrolyte. The incorporation of rGO@S composites into LGPS resulted in enhanced electronic and ionic conductivities, as well as a reduction in the cathode stress and strain and the diffusion length of Li-ions. ASSLSBs documented using this methodology exhibited an exceptional initial discharge capacity of 1629 mAh g^−1^ under a current density of 0.05 C and at a temperature of 60 °C. This development illustrates the possibility that sulfur-coated rGO composites could be utilized in ASSLSBs to improve battery performance.

Despite possessing a significant theoretical specific capacity, the electrical and ionic conductivities of elemental S and its byproduct, Li sulfide, are less than ideal, measuring around 5 × 10^−30^ and 10^−13^ S cm^−1^, respectively [159, 160]. Scientists have been motivated to enhance the electrochemical performance of ASSLSB cathode active materials in response to this constraint. Composite cathodes, which are designed to overcome this difficulty, often incorporate a high concentration of conductive carbon materials and SSEs. While this supplement enhances overall conductivity, reported studies often note a decrease in the S content. Unfortunately, this decrease in S content substantially reduces the energy density of the ASSLSBs. Researchers have devoted considerable effort over the last decade to enhancing the utilization efficiency of S materials in ASSLSBs.

#### Metal Sulfide

S-based materials have lower electrical conductivity and slower diffusion rates of Li-ions compared to metal sulfides. Consequently, they serve as cathode-active materials in ASSLSBs [156]. However, due to their larger molecular weight, the energy density of a battery made entirely of metal sulfide is not equivalent to that of elemental S. Therefore, scientists are exploring a balance between energy density and electrochemical performance by combining metal sulfides with elemental S [57, 73, 161]. In a study on cathodes for ASSLSBs, Hosseini et al. [162] investigated three separate copper sulfide-sulfur-carbon (CuSS) composites. According to their research, the composite cathode’s redox characteristics were significantly affected by the copper-sulfide-carbon (CuS/C) ratio. Among the studied composites, CuSS (2 − 1) showed the best balance, with capacities of 1200 mAh g^−1^ at 20 mA g^−1^ and 1100 mAh g^−1^ at 40 mA g^−1^, respectively. Xu et al. [155] explored metal sulfides with an intercalation type as potential cathode active materials. They developed a hybrid cathode incorporating conversion-type sulfur and intercalation-type VS_2_ to fabricate high-performance sulfide-based ASSLSBs. The battery exhibited an approximate S consumption of 85% and a reversible capacity of 1444 mAh g^−1^ (or 640 mAh g^−1^ depending on S and VS_2_) with an active material charge of 1.7 mg cm^−2^ (Fig. 4c) [155]. Additionally, a consistent areal capacity of 7.8 mAh cm^−2^ was achieved with an active material concentration of 15.5 mg cm^−2^. Kim et al. [156] conducted a comparative analysis of the electrochemical reactions involving SnS materials in both solid-state and liquid batteries (Fig. 4d). The study reported a capacity of 629 mAh g^−1^ in SnS-based solid-state batteries after 100 cycles, with a relatively small deterioration of 8.2% in the first cycle. However, during the first cycle, liquid batteries showed a significant irreversible capacity loss of 44.6%.

#### Organic Sulfur

S atoms are covalently bound to the organic framework in organic sulfur compounds, which mostly include S chains and organic components. This configuration ensures that S is uniformly distributed, preventing aggregation and thereby increasing the amount that can be utilized. Furthermore, the organic framework has the capability to mitigate the extent of enlargement that occurs during charging or discharging of the material [163, 164]. Jiag et al. [169] developed a dense composite S-carbon (S/C) cathode reinforced with a macroporous carbon (MaPC) conductive matrix (SPAN@MaPC) [165]. The reversible capacity of cells containing about 1 mg cm^−2^ of S was 1396.2 mAh g^−1^ at a rate of 0.1 C, and the capacity remained at 715.5 mAh g^−1^ after 200 cycles. The identification of a C–Li bond peak in the discharge product via X-ray photoelectron spectroscopy (XPS) suggests that irreversible C–Li bonding results in supercapacity that surpasses theoretical values.

Research into organic S-cathode materials for sulfide-based ASSLSBs is a continuous endeavor, expanding beyond sulfurized polyacrylonitrile materials. The sulfurized alcohol composite (SAC) material showed enhanced initial coulombic efficiency (ICE) and a significant specific capacity (600 to 800 mAh g^−1^) when used in ASSLSBs [166]. The use of X-ray absorption near edge structure (XANES) in subsequent studies verified that SSEs had a role in the partial lithiation of the SAC cathode while it was being ball-milled. In a related development, Yang et al. [157] introduced PTTCA as the inaugural organodisulfide cathode designed for ASSLSBs (Fig. 4e). The battery showed a 410 mAh g^−1^ reversible capacity, 767 Wh kg^−1^ energy density, and 83% capacity retention after 100 cycles when PTTCA was combined with carbon nanotubes and sulfide electrolyte.

#### Lithium Sulfide

As the completely discharged product of S, Li_2_S offers numerous irreplaceable benefits over other cathode active materials. Initially, the mitigated effect of volume change is evident when employing Li_2_S as the cathode material, given that Li_2_S represents the least dense phase with integrated Li and remains non-expansive during cell operation [167, 168]. Moreover, the energy density of the battery can be elevated through the utilization of Li_2_S as the cathode material in conjunction with a Li-free anode [169, 170]. However, Li_2_S cannot be used in ASSLSBs due to its low electrical conductivity and large activation energy barrier. These two characteristics remain barriers. To overcome the challenges inherent in the mentioned approach, researchers have been exploring the potential utilization of innovative composite cathodes composed of nanoscale Li_2_S uniformly distributed within an electronic/ionic conductive network, encompassing carbon and sulfide electrolytes. Coprecipitation and high-temperature carbonization were the two methods that Han and his colleagues employed to develop a mixed-conducting Li_2_S nanocomposite (Fig. 4f) [158]. This nanocomposite featured nanosized Li_2_S and Li_6_PS_5_Cl equally dispersed throughout the carbon matrix. Thanks to the nanoscale and uniform dispersion of carbon, Li_2_S, and Li_6_PS_5_Cl, the resulting nanocomposite demonstrated outstanding electrochemical performance. The battery maintained its capacity over an extended period and exhibited a remarkable reversible capacity of 830 mAh g^−1^ over 60 cycles at room temperature, with a Li_2_S loading of 3.6 mg cm^−2^ at 0.18 mA cm^−2^.

The possibility to achieve enhanced surface loading is presented by cathode materials based on Li_2_S, in addition to facilitating high surface loading, specific surface capacity, and capacity retention. In their study, Yan et al. [171] have shown that a Li_2_S@C nanocomposite may be created in-situ when Li metal is reacted with CS_2_. A conductive carbon matrix with uniformly implanted Li_2_S nanocrystals was revealed by transmission electron microscopy (TEM) examination. Featuring a high capacity, increased rate capability, and cycle stability, the Li_2_S@C nanocomposite cathode showcased outstanding electrochemical performance thanks to its unique architecture. The battery was found to have a capacity of 1186 mAh g^−1^ and an impressive reversible capacity of 1186 mAh g^−1^ at 0.2 mA cm^−2^, with 1.75 mg cm^−2^ of Li_2_S loads (Fig. 4g) [158]. The battery maintained an outstanding 93% capacity retention after 700 cycles, even when subjected to a high current density of 2 mA cm^−2^. This performance is especially remarkable considering the enhanced current density. Furthermore, it was possible to achieve a very high areal Li_2_S loading (7 mg cm^−2^) and a significant amount of Li_2_S consumption (91%, which is comparable to a reversible capacity of 1067 mAh g^−1^) simultaneously. Wang and his team found that, a Li_2_S@NC composite, which is a nitrogen-doped-carbon (NC)-covered Li disulfide, was formed during the pyrolysis process [172]. Enhanced rate capability, cycle stability, high reversible capacity, and 100% coulombic efficiency were some of the outstanding electrochemical features displayed by the resulting Li_2_S@NC composite. With a high capacity of 1052 mAh g^−1^ and 91% capacity retention after 50 cycles, the study demonstrated outstanding electrochemical performance in an enlarged inquiry. The significant increases in Li_2_S content (43% and 8.2 mg cm^−2^, respectively) and areal Li_2_S loading allowed for this accomplishment. Furthermore, cyclic voltammetry experiments showed that the nitrogen-doped carbon layer could speed up the redox reaction and improve Li^+^ transfer.

### Anode Materials for All-Solid-State Lithium–Sulfur Batteries

#### Lithium Metal Anode

Li metal is widely recognized as the foremost among anode materials for Li batteries, owing to its low density (0.59 g cm^−3^), the most negative voltage (− 3.04 V vs. standard hydrogen electrode (SHE)), and an exceptionally high theoretical specific capacity (3860 mAh g^−1^) [173]. The synergistic presence of these three attributes establishes Li metal as the optimal choice for anode material in Li batteries. The substantial reactivity and absence of a dedicated host in Li metal significantly constrain its applicability in ASSLSBs. When evaluating the electrochemical stability of an electrolyte, conventional methodology entails utilizing the electrochemical stability window. Upon the electrolyte interacting with Li metal and the chemical potential of the Li anode surpassing that of the lowest unoccupied molecular orbital (LUMO) or the conducting band of the SSEs, the interface experiences thermodynamic instability, precipitating spontaneous reactions with the Li metal. The electrochemical stability window for the majority of sulfide and thiophosphate electrolytes lies within the range of 1.7 to 2.3 V [174, 175]. An interaction between Li metal and the intrinsically weak P–S and M–S bonds (where M stands for Si, Sn, Ge, or Al) resulted in the creation of Li_2_S, Li_3_P, Li–M alloys (where M stands for Si, Sn, Ge, or Al), and LiX (where X stands for Cl, Br, or I). Interactions like these cause polarization and interfacial impedance to rise sharply [176, 177]. The inherent lack of a designated host for Li allows its deposition at arbitrary locations on the electrode surface, and the potential for significant volume changes in a Li anode is virtually limitless [178]. The Li/SSEs have surface defects such as voids, pores, cracks, and protrusions, all of which contribute to an uneven distribution of current. Due to the local deposition of Li, protrusions are formed, leading to an occurrence known as the “tip effect.” According to these phenomena, the more prominent a region is on the surface, the faster Li ions may be absorbed, as the intensity of the surface field disperses more widely in such areas [179] The “tip effect” exacerbates the asymmetry of electric fields, speeding up the process of dendritic development. It is expected that SSEs with a high Young’s modulus will effectively hinder Li dendrite development and penetration. However, Li dendrites tend to form readily at the interface defects between Li and SSEs. Even at low current densities, they propagate toward the pores and grain boundaries, ultimately causing rupture of the SSEs in ASSLSBs [180]. The mechanical and electrochemical properties of Li metal are intrinsically intertwined with the development of dendrites. The “creep” characteristic of Li metal, influenced by factors such as stress, current density, temperature, and particle size, significantly influences the morphology of Li deposition in ASSLSBs [181].

#### Lithium-Alloy Anode Materials

In the course of cycling, there is a recurrent observation that Li-based alloys act as a protective layer, enhancing interfacial adhesion and facilitating uniform Li plating and stripping. This phenomenon has been extensively investigated [182]. Despite the commendable specific capacity of these materials, the utilization of Li-based alloys for use as ASSLSB anode materials poses several scientific and practical challenges. Owing to its considerable potential (∼0.622 V against Li/Li^+^), which spans a wide stoichiometry range, and heightened compatibility with SSEs, the utilization of Li–In alloy as an anode material is prevalent in ASSLSBs incorporating sulfide SSEs due to its wide stoichiometry range [183]. According to Park et al. [184], the In layer does not exhibit dendritic development and encourages the construction of a stable interphase with sulfide SSEs. This allows for long-term cycling to occur without the risk of cell collapse. Conversely, Luo et al. [185] observed that elevated current density and area capacity resulted in the formation of Li–In dendrites in sulfide electrolytes. The ensuing volume shift and modest interfacial interaction were found to induce the production of Li–In dendrites that encapsulated electrolyte particles. After a protracted cycle, this eventually led to short circuits and the collapse of the cell. Moreover, due to the elevated molar mass of the Li–In alloy, there is a substantial increase in the anode weight, thereby constraining the alloy’s utility in high-energy–density ASSLSBs. Conversely, the Li–Al alloy, distinguished by a moderate potential range (0.3 ∼ 0.4 V vs. Li/Li^+^) and lower molar mass, exhibits potential in suppressing the formation of Li dendrites [186]. This property extends the longevity of the Li/SSE interfaces and facilitates the creation of batteries with enhanced energy density. Notably, in contrast to the volumetric variations observed in the Li–Si alloy (320%) and Li–Sn alloy (265%) during the lithiation and delithiation processes, the Li–Al alloy demonstrates a significantly reduced volumetric variation [187].

Silicon, an additional anode material, is widely recognized for its substantial capacity of 4200 mAh g^−1^, rendering it an outstanding selection for ASSLSBs [188, 189]. Tan and group [188] investigated anodes made of silicon with high charge capacities enabled by sulfide SSEs. The absence of carbon at the anode interface facilitated the creation of a stable SEI, effectively impeding the reduction of sulfide SSEs. The sustained operation of an anode composed of 99.9 wt% micro silicon and sulfide SSEs, is facilitated by the intrinsically low ionic and electron conductivities inherent to pure micro-silicon. The application of an external stacking pressure of 50 MPa effectively manages the gap resulting from volume expansion during the lithiation/delithiation processes of the Li–Si alloy. This methodology preserves exceptional contact between the SSEs layer and the porous structure of the delithiated Li–Si. As a result, the entire cell exhibits prolonged cycle and calendar lives while operating at a rapid rate of 25 mg cm^−2^ and a substantial current density of 5 mA cm^−2^, encompassing a wide temperature range of − 20 ∼ 80 ℃.

## Challenges Redefined in All-Solid-State Lithium–Sulfur Batteries

For potential use in EVs, ASSLSBs technology has drawn interest as a secure, durable, energy-dense (theoretically 2600 Wh kg^−1^), and cost-effective power source [87]. A surge in research endeavors in this domain was recently instigated by the discovery of SSEs exhibiting exceptional ionic conductivity. SSEs have garnered significant attention among various types, such as those composed of oxides, sulfides, polymers, and their combinations, due to their exceptional ionic conductivity at room temperature, analogous to that of liquid electrolytes [89]. Moreover, the excellent malleability of sulfide SSEs makes them suitable for reducing the interfacial impedance between particles, thus eliminating the requirement for sintering at high temperatures [90]. Particularly, the capacity of inorganic SSEs to inhibit the dissolution of polysulfides gives ASSLSBs the potential to attain greater energy density and an extended lifespan compared to traditional Li–S batteries that utilize non-aqueous liquid electrolytes [92]. Notwithstanding significant strides in the advancement of ASSLSBs, their pragmatic commercialization is impeded by several foundational challenges.

### Interface Stability

**Fig. 5:**
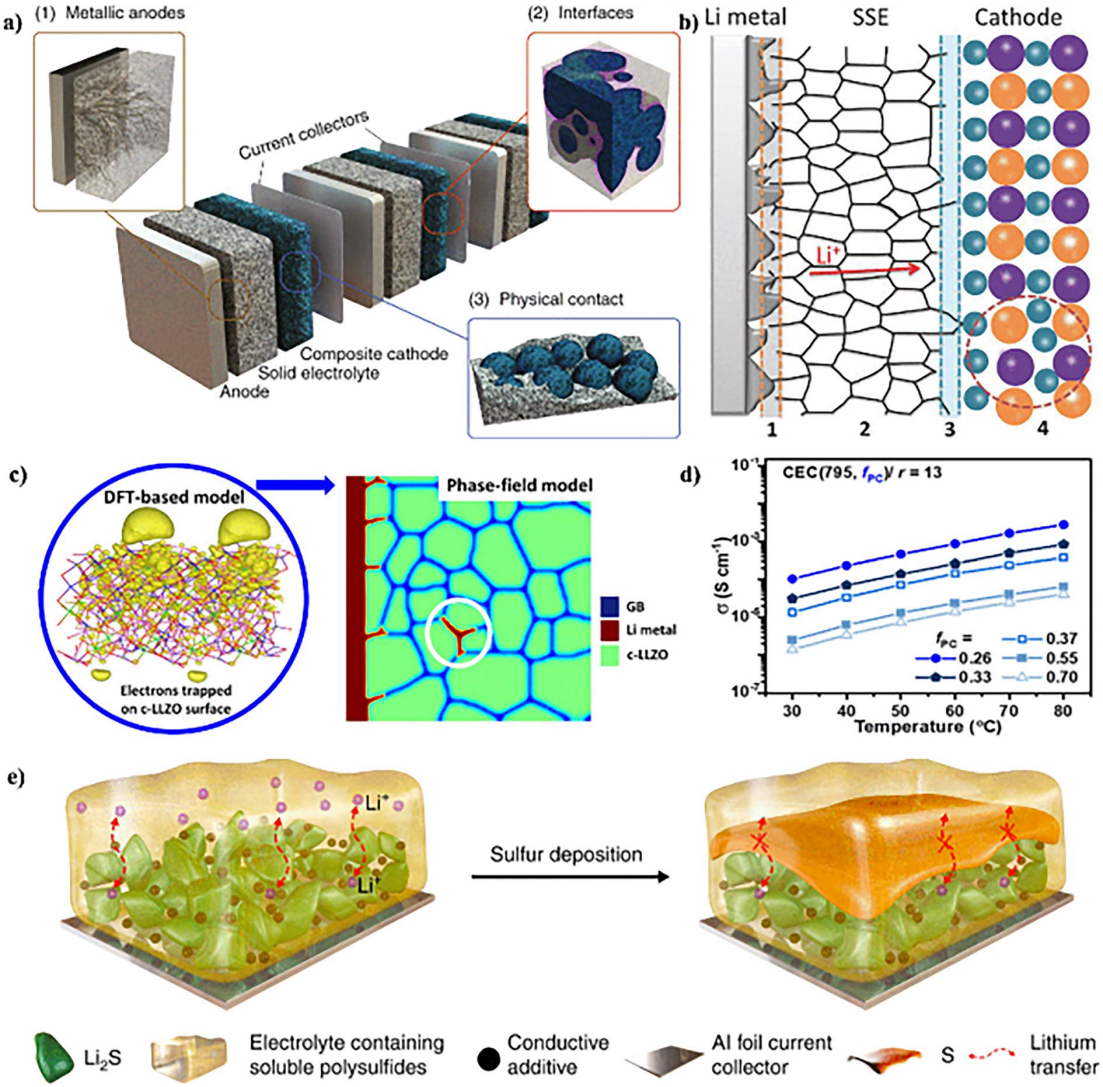
**a** Schematic representation of a bipolar-stacked solid-state battery cell. Copyright 2020, WILEY–VCH [190]. **b** Schematic diagram of the main restrictions existing in ASSLSBs by using SSEs as the electrolyte. Copyright 2018, WILEY–VCH [104]. **c** Li dendrite growth morphology in polycrystalline LLZO. Copyright 2019, American Chemical Society [193]. **d** Varying PC volume fraction (f_PC_) at a fixed PEO (n = 795) and salt ratio (r = 13). Copyright 2022, American Chemical Society [196]. **e** Sulfur deposition and the resulting blocking cause the failure of a high-loading sulfur cathode. Copyright 2022, The Authors. Published by Springer Nature [199]

Despite notable progress in achieving high bulk ionic conductivities in SSEs, the substantial impedance at the interface between the SSEs and the electrode frequently undermines the commendable conductivity achieved in the bulk material [102]. Diverging from LIBs that employ a liquid electrolyte interfacing with a solid–liquid interface, the electrochemical reactions in ASSLSBs occur at the interface established between the solid–solid electrolyte and the electrode (Fig. 5a) [190]. This interface plays a pivotal role in the operational dynamics of the battery, regulating the ion flux between the electrode and the electrolyte—a process critical to the battery’s overall functionality. An unstable interface may give rise to undesired byproducts, impede ion mobility, and ultimately contribute to the degradation of the battery’s efficiency over time. The interface between the SSEs and electrode materials is of paramount importance in shaping the electrochemical performance of ASSLSBs. The SSEs act as a barrier, preventing direct contact between the electrodes while facilitating the movement of Li ions. However, the interface may be susceptible to deterioration due to factors such as mechanical strain, chemical interactions, and differences in thermal expansion mismatch [102].

Electrode materials and SSEs interact at the interface, encompassing both hidden internal interfaces within SSEs and electrodes as well as planar interfaces between electrodes and SSE separators or current collectors [106]. Interfacial issues may arise from the direct interaction between the anode composed of Li metal and SSEs. These challenges persist not only throughout charge–discharge cycles but also during the resting state, attributed to inevitable side reactions occurring between the anode and electrolytes due to the remarkably high reducibility of Li metal [107]. These reactions generate a versatile interfacial layer, serving to partition the Li metal anode from the electrolyte [108]. An ideal interfacial layer, ensuring a Li^+^ pathway and thermodynamic stability for the electrolyte and metal anode, should be ionically conductive, electrically insulated, and resistant to electrolyte decomposition [191]. The electrochemical stability of SSEs is evidenced by their stability window. A robust electrolyte exhibits a broad stability window, with the lower limit extending beneath the Li^+^ reduction potential and the upper limit surpassing the Li^+^ extraction potential from the cathode [109]. However, due to a restricted electrochemical stability window, SSEs may rapidly decompose during cycling, causing the formation of an interfacial layer [110]. While certain interfacial layers generated via SSE decomposition may offer benefits, they are typically overly thick, leading to heightened interfacial resistance and diminished battery performance.

Sulfide-derived SSEs face substantial interfacial obstacles throughout the process of contact formation and battery functioning (Fig. 5b) [192]. One significant issue with sulfide-based cathodes is the simultaneous occurrence of chemical inter-diffusion and interfacial decomposition when exposed to high potential during each charging step. This leads to the production of insulating byproducts at the interfaces, a major contributor to increased interfacial impedance. Regardless of SSE categorization, contact loss and delamination at the interfaces between the active material and SSEs are common during battery cycling. This is attributed to volumetric fluctuations within the active material from Li intercalation and de-intercalation processes [111]. SSE decomposition may also occur at interfaces between the current collector and SSE, as well as carbon and SSE, where the SSE is exposed to active Li or Na potential [112]. While ion or electron translocation across these interfaces is not critical for battery function, this degradation undermines the SSE’s enduringly high ionic conductivity.”

### Li Dendrite

The expansion of Li dendrite and its infiltration through SSEs is a primary concern that needs resolution for the commercialization of ASSLSBs (Fig. 5c) [193]. Conventional SSEs, such as garnet Li_7_La_3_Zr_2_O_12_ and sulfide electrolytes, possess significant ionic conductivity and Li^+^ transfer rates. However, recent findings have indicated that Li dendrites may still infiltrate the electrolyte [113]. Elevated interfacial resistance, antagonistic interface reactions, grain boundary defects, and significant electronic conductivity contribute to the proliferation of Li dendrites, provoking a partial electrical short-circuit in SSEs. According to recent research, the critical current density at which dendrite perforation causes a battery to malfunction is less than 0.9 and 1.0 mA cm^−2^ for SSEs composed of oxide and sulfide electrolytes, respectively [116]. Conversely, under typical circumstances, liquid-based batteries can attain a range of 4–10 mA cm^−2^. The unexpected result of this study not only calls into question the preconceived notion that ASSLSBs are less hazardous than liquid-electrolyte batteries but also poses a conundrum for the initial progress of inorganic SSEs.

Dendrite growth in ASSLSBs can be attributed to a variety of factors [118]. Primarily, a deficiency in the interface between the SSEs and the Li-metal anode may give rise to the formation of openings, facilitating unobstructed dendrite expansion. Secondly, the low compaction density of SSEs resulting from manufacturing technologies can also create openings and promote dendrite expansion. Thirdly, conductivity disparities between the crystal and crystal boundaries of SSEs can result in the preferential deposition and dissolution of dendrites at crystal boundary sites. Lastly, the pliable phase in composite electrolytes, typically composed of polymers, can facilitate dendrite expansion compared to the mechanically robust inorganic SSEs. The proliferation of Li dendrites in ASSLSBs is highly aided by these factors, thereby posing substantial obstacles to harnessing the ‘bottleneck’ for SSE applications [119, 120]. The phenomenon of Li dendrite growth in ASSLSBs can be delineated into two distinct stages: dendrite nucleation and dendrite propagation [121]. These dendrites exhibit vulnerability to nucleation at specific sites along the interface connecting Li ions and SSEs, or alternatively, within the bulk of SSEs. The specific location of dendrite nucleation is contingent upon the surface chemistry, mechanical attributes, and electronic as well as ionic transport properties inherent to the SSEs [122]. Regarding dendrite propagation, Li dendrites typically traverse through structural imperfections such as grain boundaries, pores, and cracks present in SSEs or at the interfaces between Li and SSEs subsequent to nucleation. As dendrites persist in their propagation and exhibit an augmented diameter, they induce localized mechanical stress, ultimately leading to the evolution of cracks and the potential degradation of SSEs [123].

Recent investigations have revealed that the limited electronic conductivity inherent to SSEs may contribute to the direct initiation and expansion of Li dendrites within their internal structures. This occurs concurrently with the gradual penetration of Li dendrites originating from the anodes [124]. In a research endeavor led by Han et al. [126], the nucleation of dendrites in three distinct types of SSEs (LLZO, Li_3_PS_4_, and LiPON) was systematically compared through the application of time-resolved operando neutron depth profiling. The scientists were able to directly observe the accumulation of Li inside the interior of LLZO and Li_3_PS_4_, indicating that reducing the electrical conductivity of SSEs is essential in preventing the growth of dendrites within them. However, this research only provided large-scale descriptions and did not separate the effects of grain boundaries. Tian et al. [127] scrutinized the nucleation and formation processes of Li dendrites within SSE by employing a multiscale model that integrated density-functional theory (DFT) calculations with the phase-field method, effectively overcoming this constraint. The results showed that the surfaces of pores or cracks have a smaller energy gap compared to the SSEs bulk, which facilitates the movement of electrons from the Li metal to the surfaces and encourages dendrite growth. This was supported by microscopic evidence from Liu et al. [128], who found that narrow band gaps at grain boundaries in SSEs, such as LLZO, lead to leakage currents, intergranular Li segregation, and eventual cell short-circuits.

### Volume Expansion and Electrochemical Instabilities

The expansion and contraction that happen when active materials are (un)charged can cause cracks to form, which makes it harder for the battery to keep going through lots of cycles [194]. Electrode materials that involve conversion chemistry, like S, generally expand a lot. When lithiated to Li_2_S, the S cathodes using the chemical reaction 16Li + S_8_ ↔ 8Li_2_S display around 80% volume changes when compared to pure S [195]. However, the rigid solid-state electrolytes (SSEs) struggle to accommodate the volume shift in sulfur, resulting in stress buildup within the composite cathode. Long-term cycling will cause mechanical fractures, including the creation of cracks. More importantly, the volume shift in the positive electrodes can propagate to other battery components, including the electrolyte layers, causing severe mechanical breakdown at the electrolyte/electrode interfaces, and directly reducing the lifespan. To address this issue, researchers commonly apply significant external pressure to enhance physical interfacial contact between various components (Fig. 5d) [196]. Unfortunately, mechanical challenges arising from massive volume fluctuations still persist, restricting large-scale practical uses at low external pressure.

Despite significant advancements in the search for SSEs characterized by high ion conductivity, several fundamental challenges impede the commercialization of ASSLSBs. Of paramount importance are issues pertaining to chemical and electrochemical stability. The prevailing environmental instability of most SSEs becomes pronounced upon exposure to O_2_ and H_2_O, leading to the generation of hazardous H_2_S and subsequent SSE disintegration [197]. Furthermore, the capacities of sulfide SSEs range from 150 to 300 mAh g^−1^, with discharge plateaus exceeding 2.0 V. Electrochemical processes initiated by sulfide SSEs occur within the operational voltage windows of ASSLSBs. Notably, certain SSEs, such as LiGPS and LiSiPSCl, exhibit reactivity with the anode (Li) even in the absence of charging or discharging processes. The presence of Li metal facilitates the facile conversion of Ti^4+^ ions in lithium aluminum titanium phosphate (LATP) and lithium lanthanum titanium oxide (LLTO) to low-valence Ti^x+^ ions, resulting in a substantial reduction in the ionic conductivity of SSEs [20]. Currently, the impact of Li de-intercalation from sulfide SSEs on ionic conductivity remains unknown. These multifaceted challenges underscore the complexity associated with bringing ASSLSBs to market viability.

### Processing Challenges

In addition to material chemistry, the development and application of ASSLSBs are complex undertakings that demand an in-depth understanding of the interfacial science between SSEs and electrodes. Furthermore, the advancement of processing technology is crucial, with the need to avoid the “bucket effect” during scale-up production (Fig. 5e) [198, 199]. In contrast to polymer-based solid-state batteries, which have been mass-produced effectively using a methodology similar to that of conventional LIB fabrication, inorganic SSE-based ASSLSBs are still in the developmental or pilot production phase. Several challenges specific to ASSLSBs impede their widespread implementation [200]. Firstly, sulfides are chemically unstable when exposed to moisture, necessitating an inert processing environment and additional cost considerations for industrial-scale production. Secondly, the synthetic procedure for sulfide SSEs conventionally involves high-energy ball milling on a small scale, imposing constraints on throughput and processability when transitioning to larger scales. Finally, SSEs exhibit suboptimal mechanical properties, characterized by low fracture toughness, thereby presenting challenges in the fabrication of thin SSE membranes.

### Regulatory Approval and Standardization

In the dynamic landscape of battery technology, ASSLSBs have emerged as a promising alternative to conventional LIBs. These advanced energy storage devices exhibit higher energy density, enhanced safety features, and accelerated charging capabilities. Currently, there is no specific framework of standards or regulations governing the production and utilization of ASSLSBs [201]. Nevertheless, they are subject to overarching safety and performance standards applicable to all batteries. Significant discrepancies emerge, particularly in the utilization of SSEs compared to liquid or gel electrolytes, introducing safety and performance subtleties that current regulations inadequately address. This variability results from individual manufacturers developing distinct processes, leading to divergences in quality, performance, and cost. The absence of standardized protocols has impeded the large-scale production and pervasive adoption of ASSLSBs.

In recognition of the need for a unified strategy, major players in the battery sector have initiated cooperative efforts to establish standards for the manufacturing of ASSLSBs. These benchmarks aim to delineate standardized manufacturing processes, materials, and performance criteria. Such guidelines empower manufacturers to ensure uniform quality and compatibility across diverse production lines. An exemplary initiative in this realm is the solid-state battery initiative (SSB), a consortium comprising prominent battery manufacturers, research institutions, and government agencies [202]. The SSB seeks to expedite the advancement and commercialization of ASSLSBs by formulating a roadmap for standardization. Through collaborative research and knowledge exchange, the SSB aims to address pivotal challenges and facilitate the seamless integration of ASSLSBs into diverse applications. The consortium’s focus on vital facets of ASSLSBs production, including electrode fabrication, electrolyte synthesis, and cell assembly, aims to define optimal manufacturing techniques and material specifications, thus enhancing the efficiency and reliability of ASSLSBs production. Additionally, the SSB endeavors to devise standardized testing protocols to evaluate the performance and safety of these advanced energy storage devices.

The pursuit of standardization is not exclusive to the SSB; other entities, such as the International Electrotechnical Commission (IEC) and the International Organization for Standardization (ISO), actively engage in formulating international standards for ASSLSBs. The United Nations (UN), under the UN Manual of Tests and Criteria, has instituted regulations ensuring the secure transportation of hazardous materials, encompassing batteries [203]. These regulations stipulate packaging requirements, labeling, and testing procedures to mitigate the risks associated with transporting ASSLSBs. Moreover, national regulatory bodies, such as the U.S. Department of Transportation’s Pipeline and Hazardous Materials Safety Administration (PHMSA) and the European Union’s European Chemicals Agency (ECHA), have implemented regulations specific to ASSLSBs. These regulations are designed to address safety concerns and ensure compliance with environmental and health standards. Such standards play a pivotal role in ensuring interoperability and facilitating the global adoption of ASSLSB technology.

The establishment of benchmarks for ASSLSB production signifies a significant stride toward unlocking the full potential of this revolutionary energy storage technology. Standardization not only fortifies the reliability and safety of ASSLSBs but also catalyzes cost reduction through economies of scale. As the industry continues collaborative efforts and innovation, anticipation points to the rapid advancement and widespread deployment of ASSLSBs in the imminent future.

## Strategies to Accelerate the Commercialization of All-Solid-State Lithium–Sulfur Batteries

### Enhance Performance

**Fig. 6:**
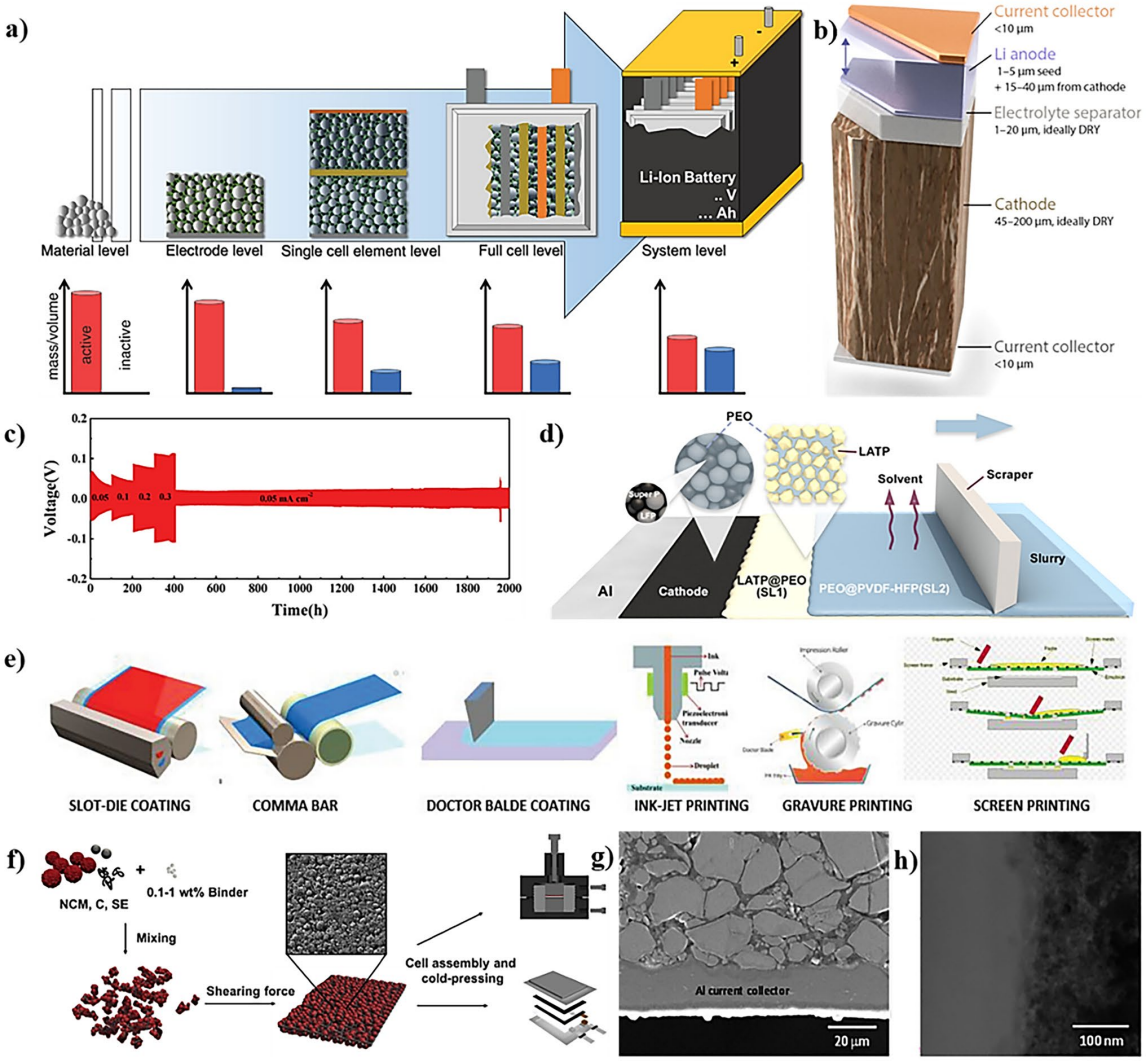
**a** Technological level to be considered during battery development and a qualitative illustration of the respective active to inactive material ratio. Copyright 2021, WILEY–VCH [204]. **b** Schematic of an ideal high-energy solid-state battery stack including a thin cathode current collector, a thick cathode, a thin electrolyte separator, a thin Li anode that expands upon charging, and a thin anode current collector. Copyright 2021, American Chemical Society [208]. **c** Long cycling performance of Li/ZnO@LATP@ZnO/Li symmetric cells at various current densities. Copyright 2019, WILEY–VCH [209]. **d** Schematic illustration of the fabrication process of the IS-CDL. ©2020, American Chemical Society [210]. **e** Sketch of different wet coating techniques used for the fabrication of solid-state batteries. Copyright 2023, WILEY–VCH [211]. **f** Schematic diagram of DF-fabrication based on a dry premixing of NCM, C, SE, and PTFE binder followed by shearing force-induced film formation. Copyright 2019 Elsevier Ltd. [212]. **g** Cross-sectional FESEM image of the LPSCl-infiltrated LCO electrode, and **h** HRTEM image of the FIB-cross-sectioned LPSCl-infiltrated LCO electrode. Copyright 2021, American Chemical Society [213]

For a precise evaluation of practical energy density, a thorough examination of all constituent elements is paramount, including, but not limited to, electrolytes, electrodes, current collectors, and cans. Furthermore, due consideration must be given to the capacity constraints of active materials. A central goal in this undertaking involves reducing SSEs to the micron scale with the aim of minimizing the presence of inactive substances (Fig. 6a) [204]. To prevent direct electrode, contact when utilizing SSEs as separators, the creation of expedient ion transport channels becomes imperative. However, the reduction in thickness, while enhancing the transport of Li ions, concurrently elevates the risk of mechanical defects culminating in short circuits [205]. Consequently, a judicious compromise must be struck in the case of SSEs to uphold their mechanical integrity while reducing their thickness. Inorganic SSEs consist primarily of oxides, sulfides, and halides. The preparation of inorganic films can be done through dry or wet processes. Although they may lack flexibility and ductility, electrolyte films with high ionic conductivity and excellent mechanical properties can be produced using both dry and wet processes [206].

For the synthesis of carbon-based SSEs, the incorporation of inorganic additives into the polymer matrix proves instrumental. Such SSEs establish enhanced contact with electrodes, exhibit superior mechanical properties, and facilitate heightened ion conductivity. This amalgamation capitalizes on favorable attributes inherent to both inorganic ceramics and polymers. Consequently, carbon-based SSEs are regarded as potentially effective components in ASSLSBs. The techniques utilized in the fabrication of inorganic ceramic and polymer films are also applicable to the production of carbon-based SSE films. Recent research has illustrated that solvent coatings anchored by ceramic polymers and chemically stable at 100 μm can be manufactured via electrospinning and solution casting. Furthermore, an innovative film composed of dual-salt-reinforced PEO/Li_6.4_La_3_Zr_1.4_Ta_0.6_O_12_ (LLZTO)/polyacrylonitrile (PAN) was fabricated through electrospinning and casting processes, yielding a remarkable ionic conductivity of 2.57 × 10^−4^ S cm^−1^ [207]. Moreover, employing the doctor blade method, a pliable ceramic/polymer composite film, tens of microns in thickness, was fabricated. The robust chemical interactions between the ceramic and polymer constituents endowed the film with a notable ionic conductivity of 9.83 × 10^−4^ S cm^−1^.

The roll-to-roll processing of carbon-based SSE films, geared towards the production of ASSLSBs, holds significant promise, showcasing exceptional ionic conductivity alongside superior mechanical properties and ductility [214].

Clearly, numerous technologies have been created to facilitate the fabrication of extremely thin electrolyte sheets. However, these films often face operational challenges at room temperature and lack the requisite mechanical strength to impede the formation of Li dendrites. Additionally, an extensive electrochemical window is necessary for coupling with cathodes operating at high voltages. As a consequence, despite extensive academic research, primarily on pouch-cell configurations for commercial use, there is still a considerable gap between laboratory findings and industrial applications. The advancement of high-energy–density ASSLSBs undeniably hinges on the judicious selection of high-performance electrodes. Solely modifying the liquid electrolyte leads to a diminution in gravimetric energy density, as SSEs inherently possess greater real densities than their liquid counterparts. Conversely, SSEs facilitate the deployment of high-capacity anodes or high-voltage cathodes impractical within liquid electrolyte systems. This capacity serves to counterbalance the external weight reduction associated with SSEs. On the anode front, electrodes characterized by higher energy densities, such as graphite and Li Titanate, are gradually giving way to those with lower energy densities. Although Li metal is considered the optimal anode for ASSLSBs, its unreliable deposition and high reactivity result in dendrite formation, rendering it impractical for application. Pure Li has a lower potential than Li alloys such as Li_x_C and Li_x_Sn, leading to a less stable interface with SSEs. Additionally, the growing prevalence of materials based on silicon can be attributed to their exceptional capacity and seamless integration with on-chip devices.

Pairing high-voltage cathodes with Li metal anodes could be an effective strategy to enhance the energy density of ASSLSBs. Extensive research has been conducted on high-Ni cathode materials, including NCM811, which demonstrates the capacity to generate an output power exceeding 200 mAh g^−1^. NRLO, characterized by an α-NaFeO_2_ structure, involves a cubic, close-packed oxygen framework with alternating arrangements of Ni and Li elements. However, Ni-rich cathodes face challenges, including intergranular cracking of grains, side reactions with SSEs, and reconstruction of surface structures when subjected to high operating voltage. While some of these materials have found practical applications, the preparation of flawless and well-crystallized particles remains a challenging task. Furthermore, in high-voltage ASSLSBs, the balance between intergranular fracture and crystalline degree must be considered. While material innovation in electrodes and SSEs can increase energy density, overall cell design optimization is necessary to reduce the occurrence of undesirable side reactions.

### Improve Cell Design to Boost Efficiency

Cell engineering comprehensively encompasses both interface engineering and the optimization of the operational conditions throughout the total cell (Fig. 6b) [208]. Of paramount importance presently is the construction of robust electrolyte interfaces between reliable electrodes, namely the anode and cathode [215, 216]. In the realm of ASSLSBs, particular emphasis is directed towards Li-metal anodes. However, an unstable SEI tends to manifest between Li anodes and SSEs. Moreover, the highly uneven deposition of Li leads to dendrite growth and penetration within SSEs, elevating the risk of short circuits. Conversely, a protective layer, requiring reaction with cathode active materials, is observed in the majority of SSEs. Consequently, the imperative to develop efficient solutions arises, aiming at fostering high-performance ASSLSBs and ameliorating electrode/solid electrolyte interfaces.

To achieve a high energy density, ASSLSBs require anodes composed of Li metal. An unstable interphase is created when Li metal, which has a strong reducing property, comes into contact with SSEs. The SEI produced by the continuous reaction of Li and SSEs is unstable. Furthermore, dendritic growth is the main cause of the brief circuits that solid-state cells develop. Currently, it is crucial to have a stable anode/SE interface, expecting the cell to operate for thousands of cycles without experiencing any short-circuiting. Determining the propensity for Li penetration is, therefore, necessary. The most efficacious approach to tackling the obstacles associated with ASSLSBs is to implement a passivated interface layer. By establishing a barrier between the electrolyte and the anode composed of Li metal, this layer prevents undesirable chemical reactions and improves stability [217]. Ideal SEIs possess characteristics such as electrical insulation, compatibility with Li metal anodes, and high Li-ion conductivity. In an effort to develop robust SEIs, various methodologies have been explored, including composite solid electrolyte (CSE) construction, atomic layer deposition (ALD), and magnetron sputtering. For example, Hao et al. [209] used magnetron sputtering to deposit an ultrathin ZnO layer onto Li_1.3_Al_0.3_Ti_1.7_(PO4)_3_ particles. A stable SEI with low electronic conductivity was generated as a result of the in-situ reactions that took place (Fig. 6c) [209]. The stable SEI hindered the growth of Li dendrites and prevented side reactions between LATP and Li metal anodes. Tang et al. [218] conducted an alternative investigation to examine interfacial resistance by fabricating an amorphous SnO_2_ layer on the surface of a Li_7_La_2.75_Ca_0.25_Zr_1.75_Nb_0.25_O_12_ garnet electrolyte via ALD. By making the interface between the garnet electrolyte and the Li metal anode, this method was able to lower resistance and completely change how well the battery worked overall. This method lowered resistance and completely changed how well the battery worked overall. In high-voltage ASSLSBs, it is essential to strike a balance between intergranular fracture and crystalline degree and to minimize undesirable side reactions via cell design as a whole [218].

The amorphous SnO_2_ layer enhances the cycle stability of Li/Garnet/Li symmetric cells by impeding garnet-Li reactions. Additionally, carbon-based SSEs can be utilized to reinforce the interface between the anode and SSE. Chen et al. [219] proposed the utilization of g-C_3_N_4_ nanosheets reinforced with polyvinylidene difluoride (PVDF) to develop a carbon-based SSE characterized by exceptional ionic conductivity and low activation energy. Furthermore, Pan et al. [214] employed in situ coupling procedures to fabricate flexible SSEs composed of polymer carbon and ceramic, enhancing interfacial compatibility and facilitating rapid ion transport. Extended cycling at ambient temperature effectively impedes the generation of Li dendrites within the symmetric cells. Hybrid cell topologies and composite anode development are additional feasible approaches to establishing stable anode/SEIs [220]. It is rare to find cathode-solid electrolyte interfaces in ASSLSBs that exhibit exceptional thermodynamic stability [221]. Moreover, the propelling force of side reactions increases substantially at high states of charge due to a combined electrochemical and chemical disintegration. The low cathode chemical potential can also drive Li extraction from the SSEs when the operating voltage is high. Following oxidation, the as-oxidized compounds may engage in a chemical reaction with the cathode material.

Different kinds of materials, including composite cathodes, CSEs, and double-layer solid electrolyte membranes, have been investigated in the search for a stable cathode/SEI. Active materials were integrated with La_2_Zr_2_O_7_ nanofibers (LZONs) and polyethylene oxide (PEO) to generate a composite cathode [222]. Anomalous “solid-polymer-solid” elastic Li-ion transport channels were a feature of this cathode. Oxygen vacancy-rich ligand-accepting regions (LZONs) not only facilitate the transportation of Li ions through efficient channels but also act as anion anchors to expedite the dissociation of Li compounds and produce a sufficient quantity of free Li ions within the PEO binder, greatly improving the efficiency of transporting Li-ions. Two SSE films composed of “polymer in ceramic” were utilized, with different amounts of PEO and LATP nanoparticles present in each film (Fig. 6d) [210]. At the cathode/SE interface, interfacial resistance can be substantially reduced when the layer has an exceptional mechanical composition and a high concentration of LATP. The high-tech Li/NCM811 batteries showed an impressive 175.5 mAh g^−1^ specific capacity at 60 °C. In addition, a PVDF-LPPO, or high-voltage ASSLSB, was created by uniformly grafting the Li phenyl phosphate (LPPO) group onto PVDF in order to create a multifunctional CSE with a branching topology [223]. By utilizing composite cathodes, multilayer structures, and CSEs, it is possible to stabilize the cells’ high-voltage cycling. Presently, the majority of cells function optimally only under conditions of elevated temperature, pressure, or current density. With thick cathodes, many batteries struggle to accomplish long and stable cycles. Improving the ionic conductivity of composite cathodes and SSEs is an excellent method for stabilizing battery operation at low pressure, ambient temperature, and high current density. In addition, cutting-edge characterization methods such as cryo-electron microscopy can assist in the comprehension of Li dendrite growth and the precarious conditions at the interface, thereby facilitating the advancement of ASSLSBs [224, 225].

### Devising Strategies for Large-Scale Fabrication

With predictions showing a surge from 160 GWh to more than 1000 GWh in the next 10 years, the worldwide battery manufacturing industry is set for significant expansion [226]. The increasing number of EVs, renewable energy systems, and portable electronic gadgets is fueling the exponential growth in demand for energy storage solutions. Resilient and cost-effective production processes are crucial to meet this growing demand and keep the business going. A significant transition from lab-scale fabrication to robust industrial production procedures is necessary to successfully integrate ASSLSBs into the market. In the initial phases of ASSLSB development, pelletized electrodes measuring several hundred micrometers in thickness were stacked. However, complex multistep pelletization is an expensive and time-consuming process that lacks scalability. An increasingly feasible strategy for the expansion of ASSLSBs would entail the creation of sheet-type electrodes that resemble those used in conventional LIBs. It is crucial to advance the development of a high-throughput manufacturing process for thin SSE films and electrodes in order to achieve this objective. The production of electrodes is inherently related to the physicochemical and electrochemical requirements of the final cell. In the near future, while metallic Li anodes are preferable due to their high energy density, graphite composite anodes may also be viable alternatives. After densification, the SSEs layer develops between the cathode and anode composites in the “SSEs-supported cell” configuration. The thickness of the SSEs layer affects the cell’s energy density since it ensures the mechanical integrity of the cell as a whole. To surmount this constraint, researchers developed a “cathode-supported two-layer cell” methodology, whereby the anode layer is stacked after the SSEs layer has been deposited onto the cathode layer.

There are numerous techniques for fabricating the electrodes in ASSLSBs. By virtue of its rapidity and capacity for expansion, the slurry coating technique distinguishes itself from the myriad alternatives when compared to the established LIB manufacturing scheme. However, incorporating SSEs into the electrode significantly modifies the solution-based manufacturing process. This matter highlights potential substitutes for moist processing. The dry film method can be effectively utilized to generate substantial cathode layers. The aerosol deposition technique (ADM) and pulsed laser deposition (PLD) were predominantly employed to deposit narrow SSE layers.

#### Wet Coating Process

As expertise acquired in the LIBs industry is readily available, the solution-based approach appears to be a feasible alternative to ASSLSBs manufacturing. A slurry containing the active ingredient, SSEs, polymeric binder, and conducting agent is initially prepared in a solvent during the wet coating process. After that, a current collector receives the slurry. The removal of the solvent results in the formation of an electrode that resembles a uniform sheet and can have its thickness fine-tuned. The primary focus for developing sulfide SSEs should be on identifying effective solvent-binder combinations. Unfortunately, due to their poor chemical stability, finding solvent-binder combinations that work for sulfide SSEs is extremely difficult, especially among those designed for LIBs [227].

The dissolution of sulfide SSEs in strong polar solvents leads to the collapse of their lattice structures, hence generating a reduction in ionic conductivity [228]. Due to this, slurries have been produced predominantly with nonpolar or weakly polar solvents. The available polymeric binders are nonpolar or less polar due to the solvent limitation. Regrettably, the current collector and electrode components adhere poorly to these binders, significantly compromising electrochemical performance and processability. The impact of interfacial resistance on rate and long-term performance is widely acknowledged, highlighting the importance of interparticle cohesiveness in ASSLSB electrodes [70]. An equilibrium between the chemical stability of SSEs and the adhesion/cohesion of binders is thus established in solution-based manufacturing.

Against this backdrop, various researchers recommended improving binder adhesion qualities and, thereby, decreasing ASSLSB resistance. This approach aligns with the prevailing consensus that the utilization of a binder can effectively impedes the chemomechanical deterioration of active materials and SSEs by effectively withstanding internal stress. The research conducted by Lee et al. [229] wherein they optimized the polarity of the binder, is crucial to this discussion. The polarity of styrene-butadiene-block-copolymers (SBS) was precisely regulated by means of a thiol-ene click reaction in which carboxylic acid was grafted [230]. The regulated polarity of the binder led to the invariance in the chemical identity of the sulfide SSEs in the low-polarity p-xylene solvent, resulting in the effective generation of significant hydrogen bonding between the polymer and the metal oxide cathode. Polarity matching was resolved in a separate investigation conducted by the same group through the application of protection-deprotection chemistry (Fig. 6e). During the slurry mixing phase, tert-butyl groups were used to protect the carboxylic acid groups of the binder. This made them less polar and better able to mix with the sulfide-based SSEs and butyl butyrate solvent. During the dehydrating process, heat cut through the protective group, revealing the polar functional groups that were connected to the metal oxide active material through hydrogen bonding. It is worth mentioning that the deprotected electrode, which contained a considerable amount of LiNi_0.7_Co_0.15_Mn_0.15_O_2_ (16 mg cm^−2^), exhibited a remarkable level of adhesion strength, as evidenced by its peeling strength of 19.5 gf mm^−1^.

Conversely, scholars have also investigated the application of binders that augment ion conductivity as a means to reduce interfacial resistance. In their study, Oh et al. [231] devised Li-ion conductive binders by combining nitrile-butadiene rubber (NBR) with a Li salt solvated in an ionic liquid. This composition facilitates the transport of ions at the interface. Due to the fluidic properties of the ionic liquid, cells utilizing this Li-ion conductive binder demonstrated greater specific capacities than those employing conventional NBR-based binders. However, viscosity, (electro)chemical stability, and flammability are all properties of viscous fluids that must be taken into account, as they can influence the rheological properties of the slurry. In solution-processed manufacturing, it is common practice to utilize a dual layer-by-layer coating. This method involves applying the coating in the following order: cathode and SSEs [232]. Due to the possibility that the SSEs solution utilized for the second coating will dissolve the electrode components and destabilize the precast cathode layer, extreme caution must be exercised when applying a dual coating.

#### Dry Coating Process

The research community has redirected its attention towards dry techniques for building LIBs and ASSLSBs, as the use of organic solvents in wet coating procedures has been found to have adverse environmental consequences [211]. Dry processes offer a viable alternative as they do not require cleanup after manufacturing. Polytetrafluoroethylene (PTFE), a fibrous material often used in substance extrusion, is one potential binder for dry techniques. A film is formed by combining dried active material, conductive additive, PTFE, and SSEs during the dry process and subsequently rolling it onto a current collector. Hippauf et al. [212] demonstrated the utilization of a dry film technique to fabricate a cathode sheet with a significant areal capacity, employing a minimal quantity of binder (Fig. 6f). Importantly, electrodes manufactured via the dry-film method demonstrated enhanced energy density performance compared to those produced through solution-based techniques. This finding suggests that the dry-film process provides energy density benefits. Nakamura et al. [233] introduced a dry coating methodology for the generation of core–shell composite particles for an all-solid-state battery. In the dry coating process, the larger core particles were coated directly with the smaller fine particles by external mechanical forces without using any solvents and binders. They successfully demonstrated the uniform coating of a single LiNi_1/3_Co_1/3_Mn_1/3_O_2_ particle with a continuous layer of Li_3_PS_4_, generating an interfacial contact area between NCM and LPS and well-percolated ion transport pathways. This method holds the potential to modernize the battery industry on an industrial scale; nevertheless, it remains a novel fabrication method necessitating further refinement and enhancement. For instance, the study of mechanical distribution among dry coating processes is incomplete. Dry coating can be achieved in many ways. Different adhesion methods cause internal binder distribution differences [234]. Conversely, the absence of a definitive evaluation of various binders in the dry coating process concerning their stability at high voltages poses a challenge. This challenge encompasses reduced coulombic efficiency attributable to additional active lithium consumption, as well as the initiation of mechanical strength degradation, compromised ionic transport, and collapse of the electrode structure [235, 236].

#### Infiltration Process

Solution infiltration is a prospective method for the fabrication of sheet-type SSE layers. The porous membrane is filled with a solution containing SSEs or SSE precursors, utilizing capillary force. Subsequently, the SSEs are formed in situ by a recrystallization process [237]. This approach facilitates the production of composite electrodes, circumventing the challenges associated with binder-solvent compatibility typically seen in SSEs. In their study, Kim et al. [237] fabricated a conventional LIBs electrode by employing a PVDF binder that was dissolved in *N*-methyl-2-pyrrolidinone. Subsequently, the electrode was subjected to the introduction of Li_6_PS_5_Cl SSEs, leading to the establishment of uniformly dispersed Li-ion pathways within the active particles (Fig. 6g, h).

Solution infiltration is a frequently utilized technique in SSEs to generate distinct layers. Owing to their remarkable mechanical strength and elasticity, self-assembling monolayers of supramolecular structures have been constructed extensively using polymeric templates as frameworks. In their study, Kim et al. [238] effectively constructed a layer of Li_6_PS_5_Cl_0.5_Br_0.5_ SSEs with a thickness ranging from 40 to 70 µm. This was achieved by infiltrating a solution of SSEs into a nonwoven scaffold made of electrospun polyimide, followed by heating the composite material to a temperature of 400 °C. Typically, liquid-phase synthesis of sulfide SSEs produces insufficient ionic conductivities, requiring further annealing treatment [239]. Thermally resistant polymers, such as polyimide are recommended as scaffolds for withstanding high-temperature annealing. Nonetheless, this heat treatment can exacerbate side effects at the active material/SSE interactions. Furthermore, insufficient SSE solution penetration or the formation of voids following solvent evaporation might raise interfacial resistance, necessitating electrode densification as an additional step [213].

## Summary and Outlook

This review article explores the promising realm of ASSLSBs as a potential successor to conventional LIBs. Recognized as a significant advancement in energy storage technology, ASSLSBs capitalize on Li–sulfur reversible redox processes, offering remarkable advantages such as superior energy density, extended operational lifespan, and enhanced safety features. Despite their immense potential, the commercial adoption of ASSLSBs has been slow, prompting the need for accelerated research and development. The article thoroughly reviews the current state of ASSLSBs, providing an in-depth analysis of the rationale behind their transition, delving into the fundamental scientific principles, and offering a comprehensive assessment of the primary challenges hindering their commercialization. The authors advocate for a strategic focus on the following key areas to expedite the deployment of ASSLSBs in the commercial sector:

The large-scale production of ASSLSBs faces a complex array of challenges, necessitating innovative strategies for successful execution. A significant impediment involves attaining uniform and scalable deposition techniques for SSEs, ensuring consistent and high-caliber interfaces among diverse components. Additionally, mitigating the intrinsic instability of sulfur electrodes and formulating effective encapsulation methods to counter polysulfide shuttling represent pivotal challenges. Furthermore, optimizing manufacturing processes to enhance energy density, cycle life, and overall battery performance on a large scale demands a comprehensive approach, incorporating considerations of cost-effectiveness, environmental impact, and safety. Addressing these intricate challenges requires a convergence of materials science, engineering, and manufacturing expertise, working together to propel the development of reliable and commercially viable ASSLSBs for the future.

To enhance the practical energy density of ASSLSBs, a critical consideration lies in reducing the proportion of inactive substances. Comprehensive estimation of practical energy density necessitates the evaluation of all battery components, encompassing electrolytes, electrodes, current collectors, and cans, along with recognizing the capacity limits of active materials. A pivotal strategy involves reducing the thickness of the SSEs to micron levels, a move that mitigates the prevalence of inactive materials. However, this reduction in thickness, essential for enhancing Li-ion transport, introduces a conundrum as it heightens the risk of short circuits due to potential mechanical failures. Striking a delicate balance between decreasing thickness and sustaining the mechanical integrity of SSEs becomes imperative to navigate this challenge successfully. The quest for optimal energy density in ASSLSBs, therefore, unfolds as a nuanced pursuit, demanding careful calibration of the thickness of SSEs to harness the benefits of enhanced ion transport while mitigating the risks associated with mechanical vulnerabilities.

The commercialization of ASSLSBs is confronted by formidable challenges intricately linked to the low performance of electrodes and high interfacial resistance. Overcoming the challenges will necessitate a multidimensional approach that incorporates advances in materials science, electrochemistry, and engineering. Firstly, extensive research and development efforts should be directed toward the design and manufacture of electrode materials with improved electrochemical properties, such as high conductivity, stability, and compatibility with SSEs. Furthermore, tuning the electrode–electrolyte interface is critical. Surface modifications, interfacial engineering, and the creation of protective coatings can all help to reduce interfacial resistance and improve overall battery efficiency. Collaborative efforts across academia, business, and research institutions are critical to fostering innovation and accelerating the development of viable solutions to these difficulties, allowing for the wider adoption of high-performance ASSLSBs in practical applications.
